# Supplementary material for: Differential ion mobility mass spectrometry in immunopeptidomics identifies neoantigens carrying colorectal cancer driver mutations
Source: Commun Biol. 2022 Aug 18;5:831. doi: 10.1038/s42003-022-03807-w (PMC9388627; doi:10.1038/s42003-022-03807-w)
Supplement: Supplementary file 2 — Supplementary Information [file 42003_2022_3807_MOESM2_ESM.pdf]

Supplementary Information

Differential ion mobility mass spectrometry in immunopeptidomics identifies neoantigens carrying colorectal cancer driver mutations

Yuriko Minegishi[1], Kazuma Kiyotani[2], Kensaku Nemoto[2], Yoshikage Inoue[3], Yoshimi Haga[1], Risa Fujii[1], Naomi Saichi[1], Satoshi Nagayama[4], Koji Ueda[1*]

Contents of Supplementary information

* Supplementary Methods
* Supplementary Figures

Supplementary Methods

Cell line samples

The following cell lines were obtained: HCT116, colorectal cancer cell line, from American Type Culture Collection (ATCC); Colo668, brain metastasized small cell lung adenocarcinoma cell line from Sigma Aldrich; and RCM1, colorectal cancer cell line, from Japanese Collection of Research Bioresources (JCRB). All cells were cultured in RPMI 1640 medium (WAKO) with 10% heat-inactivated fetal bovine serum (FBS), penicillin G and streptomycin and maintained under general conditions. Cells were washed once with PBS before harvest. The desired number of cell pellets was stored at -80 ℃ until use.

In-house purification of antibody and IP bead preparation

A hybridoma clone (HB95) of anti-panHLA alpha chain antibody (W6/32) was obtained from ATCC. Hybridoma cells were first expanded in DMEM containing 10% FBS and then cultured in CELL Line flasks (WHEATON) with COS medium (COSMO BIO) for antibody production. After 1 week of cultivation, the culture media was collected and affinity purified by Protein A Fast Flow (GE). Small aliquots of eluates were electrophoresed and then stained with GelCode Blue Stain Reagent (Thermo Scientific) to check IgG fractions. The IgG-enriched fractions were pooled and then dialyzed to replace the buffer with PBS. The antibody concentration was examined by BCA assay. To confirm the sufficient affinity of our in-house purified W6/32 antibody against HLA molecules, we tested all batches of W6/32 by general IP and Western blotting with an antibody for anti-pan HLA class I α-chain molecules (clone EMR8-5, MBL) and b-2-microglobulin (b2M) antibody (PROTEINTECH) in advance. High affinity confirmed that the W6/32 antibody (800 µg) was then cross-linked onto 200 µl of a slurry of Protein G Sepharose (GE) by DMP (20 mM) in HEPES (pH 8.4) after 1 hour. After quenching with TBS-T for 1 hour, beads were used for IP or stored at 4 ° C before use.

Immunoprecipitation of HLA-Class I Complex and HLAp purification for Immunopeptidomics

The desired cell pellet was lysed in 1 mL of lysis buffer containing [20 mM] HEPES, [150 mM] NaCl, 1% NP-40, 0.1% SDS and 10% glycerol on ice. Iodoacetamide at a final concentration of 0.2 mM and protease inhibitor cocktail (Halt™) were added to the lysis buffer just before lysate preparation. For tissue samples, a Bio Masher System disposable homogenizer (nippi) was used first with 500 µl of lysis buffer for approximately 40 mg of tissue on ice, and then another 500 µl of lysis buffer was added to make the sample volume 1 mL. Both cell and tissue samples were cleared by centrifugation at 4 ° C and 15,000 rpm for 15 min, and the supernatant was collected and subjected to immunoprecipitation at 4 ° C overnight on a rotating rotor. For thorough washing, a Bio Spin Column (BioRad) was used afterward. The washing steps were as follows: 1 mL of lysis buffer 5 times, 1 mL of PBS-T 5 times, 1 mL of PBS 5 times, 1 mL of high-salt PBS twice, 1 mL of PBS 5 times and then 1 mL of mass spectrometry-grade water twice. The trimer of the HLA complex was dissociated and eluted from IP beads with 500 µl of 1% TFA. The obtained HLA-enriched eluate was further processed by tC18 SepPak. For class I HLA peptides, 500 µl of 20% acetonitrile (ACN) was first used for elution. HLA peptide-containing eluates were then dried by an evaporator. To elute the other components of the HLA complex to confirm the IP efficiency, 500 µl of 80% ACN was used for the second elution, and the obtained eluates were dried by an evaporator. The eluates of 20% ACN were analyzed by mass spectrometry as immunopeptidomics samples. The eluates of 80% ACN were used to monitor the IP efficiency by Western blotting, and sample purity was determined by silver staining (Silver Quest).

Differential Ion Mobility (DIM) Mass Spectrometry (MS) by High-Field Asymmetric-waveform Ion Mobility Spectrometry (FAIMS) for Global Immunopeptidomics

The sample including HLAp was first trapped by a precolumn (C18 Accleim PepMap 100 C18 Trap Cartridge, Thermo Scientific). The trapped sample was separated by an analytical column (Aurora UHPLC Column, C18, 0.075 × 250 mm, 1.6 µm FSC, ESI, IonOpticks) coupled with a nanospray Flex ion source for electrospray ionization (Thermo Scientific). The Ultimate 3000 RSLC nano HPLC system was used at a flow rate of 200 nl/min using a linear gradient starting from 2% to 28% solvent B (0.1% formic acid in acetonitrile) over 55 min, followed by a 1 min hold at 95% solvent B prior to a 1 min analytical column equilibration with 2% solvent B. We used 0.1% formic acid in water as solvent A. To validate the benefit of FAIMS-applied DIM-MS in immunopeptidomics, conventional liquid chromatography tandem mass spectrometry (LC–MS/MS) and LC-FAIMS-MS/MS conditions were first compared. For LC-FAIMS-MS/MS, the FAIMS-Pro interface (Thermo Fisher Scientific) was installed onto an Orbitrap Fusion Lumos Tribrid mass spectrometer and operated with default parameters except for the compensation voltage (CV) settings for gas-phase fractionation. For LC–MS/MS, we uninstalled the FAIMS device from the mass spectrometer and performed analyses. Under LC-FAIMS-MS/MS conditions, the sample was seamlessly fractionated by 3 CVs per single analysis. A total of 3 CV sets were used per sample, and each CV set included the following: set 1 (CV = -40, -60, -80 V), set 2 (CV = -35, -50, -70 V) and set 3 (CV = -45, -55, -65 V). The cycle time was optimized to 1 sec per CV to accommodate three simultaneous CVs within a single analysis. Therefore, a total of 3 analyses (raw data) that included 9 CVs were acquired per sample. The parameters for mass spectrometry were optimized for immunopeptidomics with a small amount of sample. Full MS (ranging from 320 to 850 m/z) in the Orbitrap was acquired at a resolution of 60k followed by an MS2 acquisition at a resolution of 15k.

Supplementary Methods

The maximum injection time for the full MS scan was 50 ms with an auto gain control (AGC) of 4e5. The maximum injection time for the MS2 scan was 100 ms with an AGC of 1e4 followed by top speed MS2 acquisition by the ion trap. Charge states 2 and 3 were selected for fragmentation by collision-induced dissociation (CID) at a rapid scan rate at 30% collision energy. The easy-IC system was used for the internal calibration lock mass during the data acquisition. The HCT116 samples were prepared from 1e8 cells and proportionally injected into the MS to match the indicated number of cells. For the analyses of 1e8 HCT116 cells and clinical tissue samples, first, 1/20 of the sample volume was used for analyses of 3 CV sets to check the sample conditions, and then, from the remaining samples, 5/20 of the sample volume was used for 3 CV sets. Therefore, a total of 6 analyses were performed per clinical tissue sample. The LC/MS raw data and summarized result files are deposited in a public proteomic database, the Japan Proteome Standard Repository/Database (jPOST), as follows: HCT116 HLAp without FAIMS in JPST001072, HCT116 HLAp with FAIMS in JPST001066, global identification of HCT116 HLAp in JPST001068, HLAp from normal regions of CRC tissues in JPST001070, and HLAp from tumor regions of CRC tissues in JPST001069.

Supplementary Figure 1

a)

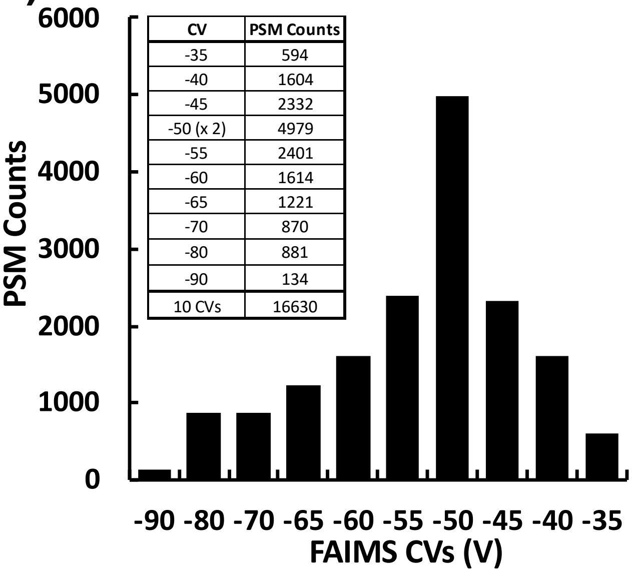

CV	PSM Counts
-35	594
-40	1604
-45	2332
-50 (x 2)	4979
-55	2401
-60	1614
-65	1221
-70	870
-80	881
-90	134
10 CVs	16630

b)

Group	3CVs			4CVs		5CVs	
CVSet	3CVSet-1	3CVSet-2	3CVSet-3	4CVSet-1	4CVSet-2	5CVSet-1	5CVSet-2
CVs	CV-40/-60/-80	CV-35/-50/-70	CV-45/-55/-65	CV-35/-48/-61/-73	CV-41/-54/-67/-80	CV-40/-50/-60/-70/-80	CV-35/-45/-55/-65/-75
MS/MS	17290	18753	19841	19017	18820	19591	19589
PSMs	3449	3705	5902	4543	440	5047	4927
**Peptide Grouops**	**3015**	**3130**	**3906**	**3261**	**3233**	**3046**	**3047**
Protein Groups	2050	242	2542	2177	2196	2070	2091
8-15aa in Length	2991	3016	3883	3242	3207	3030	3053
Deduplicated	2892	3002	3747	3129	3111	2935	2942
# of No-binder by MHCpan	77	77	101	85	72	59	75
**Confirmed HLAp**	**2815**	**2925**	**3647**	**3044**	**3039**	**2876**	**2867**
CVSet 1 + CVSet2	3896			3892		3623	
CVSet 1 + CVSet3	**4331**			n.a.		n.a.	
CVSet2 + CVSet3	4258			n.a.		n.a.	
CVSet1 + CVSet2+ CVSet3	**4742**			n.a.		n.a.	

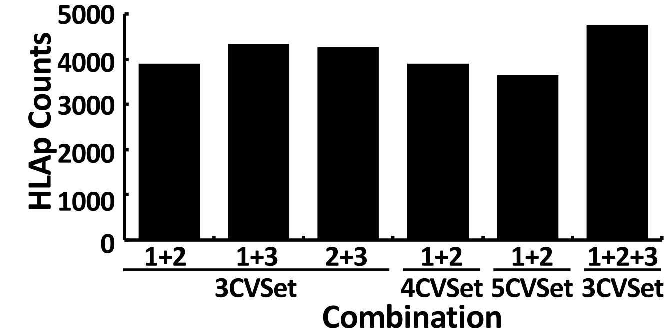

Supplementary Figure 1. FAIMS CV-distribution and optimal combination of CV set for immunopeptidomics by FAIMS-assisted DIM-MS. a) First, we roughly checked the CV distribution of immunopeptides by PSM counts to select optimal CVs for DIM-MS. PSMs of immunopeptide samples were mainly distributed in the CV range from -80 V to -35 V. Within this range, the CV range from -55 to -45 V occupied almost 50% of total PSMs. This trend was sample-unique and could slightly oscillate accordingly due to the possible differences in the genetic background of HLA allotypes in each sample. **b**). Based on the CV distribution of the immunopeptide shown in **a)**, we next checked ID efficiency by 3 CVs/run, 4 CVs/run and 5 CVs/run in combination. To accommodate multiple CVs/run, we set the cycle time to 1 sec for each CV experiment. As a result, 3CVsets x 2 runs identified the immunopeptide more efficiently when compared to the 4CVset x 2 runs or the 5 CVset x 2 runs. The extra run of the sample, *i.e.*, 3CVsets x 3 runs, further expanded the identification efficiency. Based on these results from pilot studies, DIM-MS by 3CVsets (cycle time of 1 sec each) x 3 runs per sample was established.

Supplementary Figure 2

a)

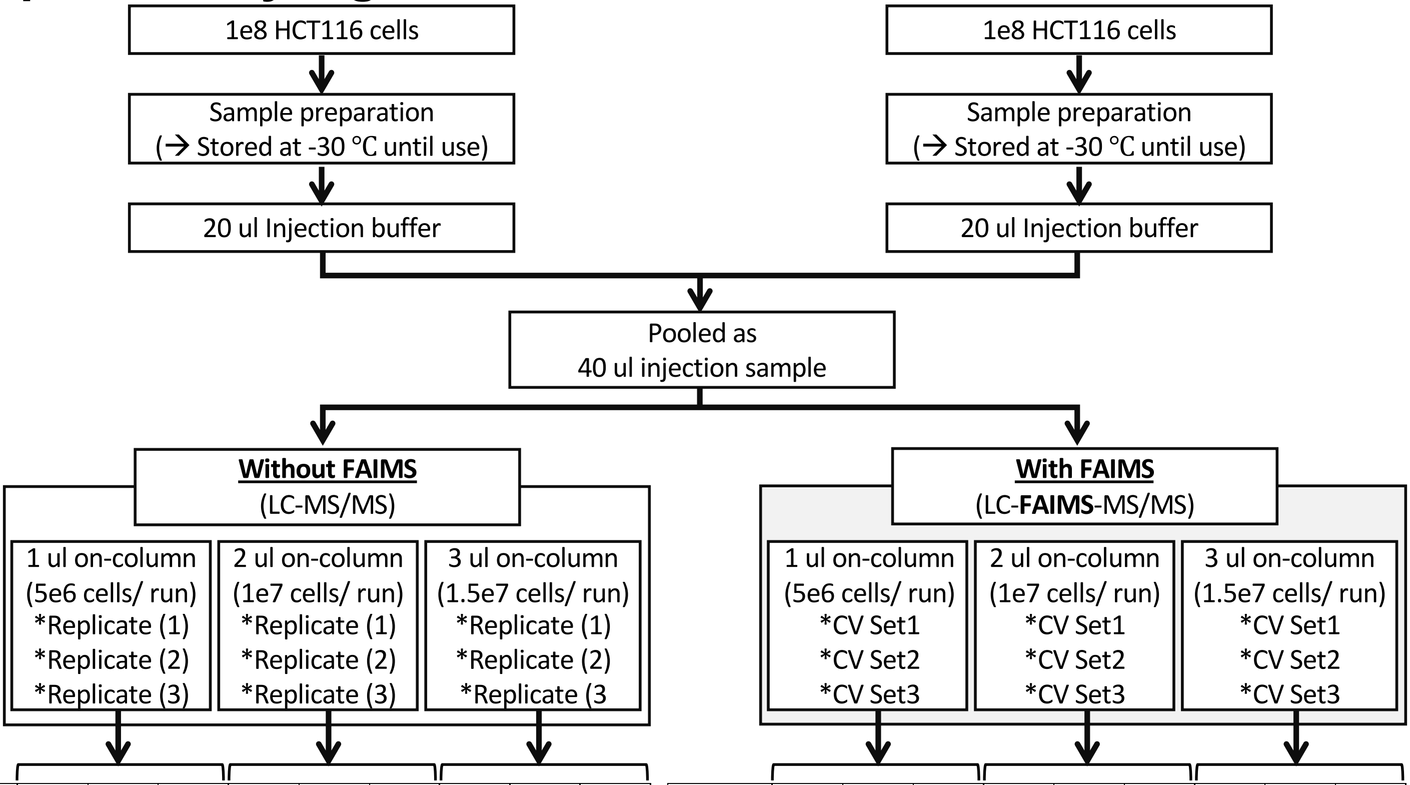

Sample	1ul-(Replicate 1)	1ul-(Replicate 2)	1ul-(Replicate 3)	2ul-(Replicate 1)	2ul-(Replicate 2)	2ul-(Replicate 3)	3ul-(Replicate 1)	3ul-(Replicate 2)	3ul-(Replicate 3)
MS/MS	14831	14947	14895	18308	17379	17224	20061	19060	19149
PSMs	3563	3405	2751	4298	3676	3545	4764	3913	3986
**Peptide Grouops**	**3215**	**3085**	**2531**	**3886**	**3366**	**3242**	**4315**	**3613**	**3640**
Protein Groups	2217	2163	1851	2556	2308	2223	2763	2440	2425
8-15aa in Length	3199	3068	2514	3859	3348	3222	4285	3585	3608
**Deduplicated**	**3107**	**2986**	**2448**	**3746**	**3255**	**3134**	**4143**	**3479**	**3516**
# of No-binder by MHCpan	86	84	55	121	94	101	134	108	112
**Confirmed HLAp**	**3021**	**2902**	**2393**	**3625**	**3161**	**3033**	**4009**	**3371**	**3404**
**Neoantigens**	**4**	**2**	**1**	**2**	**1**	**2**	**2**	**1**	**2**
**Details of Neoantigen**	CHMP7-A324T	CHMP7-A324T	NAPA-A181V	NAPA-A181V	NAPA-A181V	NAPA-A181V	CHMP7-A324T	NAPA-A181V	NAPA-A181V
	GAPDH-I69T	NAPA-A181V		AGO2-H336Y		FNBP4-K318E	NAPA-A181V		CHMP7-A324T
	NAPA-A181V								
	NR1D1-G39D								
**Merged: Confirmed HLAp**	Merged		**3986**	Merged		**4681**	Merged		**5103**
**Merged: Neoantigens**	Neoantigen		**4**	Neoantigen		**3**	Neoantigen		**6**
**Total**	**Merged**								**6065**
**Neoantigens**	**Neoantigen**								**6**

**Sample**	1ul-3CVsSet1	1ul-3CVsSet2	1ul-3CVsSet3	2ul-3CVsSet1	2ul-3CVsSet2	2ul-3CVsSet3	3ul-3CVsSet1	3ul-3CVsSet2	3ul-3CVsSet3
**No-binder Rate (%): (No-binder/ Peptide Groups)**	2.67	2.72	2.17	3.11	2.79	3.12	3.11	2.99	3.08
	Average		2.52	Average		3.01	Average		3.06
	**Average**								**2.86**

**Sample**	1ul-3CVsSet1	1ul-3CVsSet2	1ul-3CVsSet3	2ul-3CVsSet1	2ul-3CVsSet2	2ul-3CVsSet3	3ul-3CVsSet1	3ul-3CVsSet2	3ul-3CVsSet3
**No-binder Rate (%): (No-binder/ Deduplicated)**	2.77	2.81	2.25	3.23	2.89	3.22	3.23	3.10	3.19
	Average		2.61	Average		3.11	Average		3.17
	**Average**								**2.97**

**Sample**	1ul-3CVsSet1	1ul-3CVsSet2	1ul-3CVsSet3	2ul-3CVsSet1	2ul-3CVsSet2	2ul-3CVsSet3	3ul-3CVsSet1	3ul-3CVsSet2	3ul-3CVsSet3
**Hit Rate (%): (Confirmed HLAp/ Peptide Groups)**	93.97	94.07	94.55	93.28	93.91	93.55	92.91	93.30	93.52
	Average		**94.19**	Average		**93.58**	Average		**93.24**
	**Average**								**93.67**

**Sample**	1ul-3CVsSet1	1ul-3CVsSet2	1ul-3CVsSet3	2ul-3CVsSet1	2ul-3CVsSet2	2ul-3CVsSet3	3ul-3CVsSet1	3ul-3CVsSet2	3ul-3CVsSet3
**Hit Rate (%): (Confirmed HLAp/ Deduplicate)**	97.23	97.19	97.75	96.77	97.11	96.78	96.77	96.90	96.81
	Average		**97.39**	Average		**96.89**	Average		**96.83**
	**Average**								**97.03**

Sample	1ul-3CVsSet1	1ul-3CVsSet2	1ul-3CVsSet3	2ul-3CVsSet1	2ul-3CVsSet2	2ul-3CVsSet3	3ul-3CVsSet1	3ul-3CVsSet2	3ul-3CVsSet3
MS/MS	17278	18955	20468	19708	19937	22163	20988	21324	23963
PSMs	3741	4052	6195	4747	5229	7232	4996	5502	7645
**Peptide Grouops**	**3265**	**3421**	**4014**	**4053**	**4385**	**4673**	**4332**	**4609**	**4898**
Protein Groups	2225	2331	2588	2637	2826	2927	2720	2915	3008
8-15aa in Length	3242	3400	3997	4024	4359	4653	4302	4587	4872
**Deduplicated**	**3146**	**3309**	**3883**	**3916**	**4245**	**4533**	**4192**	**4459**	**4744**
# of No-binder by MHCpan	84	69	88	108	94	114	117	117	118
**Confirmed HLAp**	**3062**	**3240**	**3795**	**3808**	**4151**	**4419**	**4075**	**4342**	**4626**
**Neoantigens**	**5**	**3**	**3**	**4**	**3**	**4**	**6**	**6**	**5**
**Details of Neoantigen**	NAPA-A181V	CHMP7-A324T	CHMP7-A324T	CHMP7-A324T	CHMP7-A324T	CHMP7-A324T	CHMP7-A324T	RBBP7-N17D	RBBP7-N17D
	NOTCH2-C41S	GAPDH-I69T	GAPDH-I69T	NAPA-A181V	NAPA-A181V	GAPDH-I69T	NAPA-A181V	GAPDH-I69T	CHMP7-A324T
	CHMP7-A324T	NAPA-A181V	NAPA-A181V	GAPDH-I69T	GAPDH-I69T	NAPA-A181V	GAPDH-I69T	CHMP7-A324T	NAPA-A181V
	GAPDH-I69T			NR1D1-G39D		IQGAP3-S1070T	IQGAP3-S1070T	NAPA-A181V	GAPDH-I69T
	FNBP4-K318E						NOTCH2-C41S	NR1D1-G39D	IQGAP3-S1070T
							NR1D1-G39D	FNBP4-K318E	
**Merged: Confirmed HLAp**	Merged		**5079**	Merged		**6083**	Merged		**6469**
**Merged: Neoantigens**	Neoantigen		**5**	Neoantigen		**5**	Neoantigen		**8**
**Total**	**Merged**								**7522**
**Neoantigens**	**Neoantigen**								**8**

**Sample**	1ul-3CVsSet1	1ul-3CVsSet2	1ul-3CVsSet3	2ul-3CVsSet1	2ul-3CVsSet2	2ul-3CVsSet3	3ul-3CVsSet1	3ul-3CVsSet2	3ul-3CVsSet3
**No-binder Rate (%): (No-binder/ Peptide Groups)**	2.57	2.02	2.19	2.66	2.14	2.44	2.70	2.54	2.41
	Average		2.26	Average		2.42	Average		2.55
	**Average**								**2.41**

**Sample**	1ul-3CVsSet1	1ul-3CVsSet2	1ul-3CVsSet3	2ul-3CVsSet1	2ul-3CVsSet2	2ul-3CVsSet3	3ul-3CVsSet1	3ul-3CVsSet2	3ul-3CVsSet3
**No-binder Rate (%): (No-binder/ Deduplicated)**	2.67	2.09	2.27	2.76	2.21	2.51	2.79	2.62	2.49
	Average		2.34	Average		2.50	Average		2.63
	**Average**								**2.49**

**Sample**	1ul-3CVsSet1	1ul-3CVsSet2	1ul-3CVsSet3	2ul-3CVsSet1	2ul-3CVsSet2	2ul-3CVsSet3	3ul-3CVsSet1	3ul-3CVsSet2	3ul-3CVsSet3
**Hit Rate (%): (Confirmed HLAp/ Peptide Groups)**	93.78	94.71	94.54	93.96	94.66	94.56	94.07	94.21	94.45
	Average		94.35	Average		94.39	Average		94.24
	**Average**								**94.33**

**Sample**	1ul-3CVsSet1	1ul-3CVsSet2	1ul-3CVsSet3	2ul-3CVsSet1	2ul-3CVsSet2	2ul-3CVsSet3	3ul-3CVsSet1	3ul-3CVsSet2	3ul-3CVsSet3
**Hit Rate (%): (Confirmed HLAp/ Deduplicate)**	97.33	97.91	97.73	97.24	97.79	97.49	97.21	97.38	97.51
	Average		97.66	Average		97.50	Average		97.37
	**Average**								**97.51**

b)

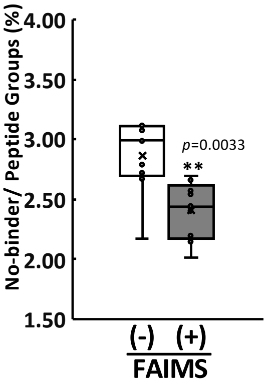

c)

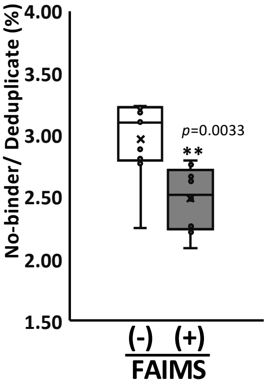

Supplementary Figure 2. Experimental validation without or with FAIMS for HCT116 immunopeptidomics. a) A schematic diagram of validation analyses and the processing are shown in Figure 1b to 1e. The pooled sample was injected for three replicates without FAIMS and compared with the three distinct CVset runs (total 9 CVs) with FAIMS. Each count was compared between the without and with FAIMS conditions. **b)** and **c)** FAIMS-assisted DIM-MS increased not only the counts of peptide groups and processed deduplicated candidates but also the purity of immunopeptide identification in each candidate. The boxplots depict a statistically significantly reduced number of nonbinders in peptide groups **b)** and after deduplication **c)**.

Supplementary Figure 3

a) HCT116

Sample preparation from 1e8 HCT116 Cells
Sample dissolved in 20ul of Injection Buffer
MS on-column volume: 5ul (≒2.5e7 cells)/ CVSet)
Total: 7.5e7 cells
Analyses: 3 CVSets → Total 9 FAIMS CVs

	Number
MS/MS	55259
PSMs	17996
Peptide Groups	6223
Protein Groups	3552

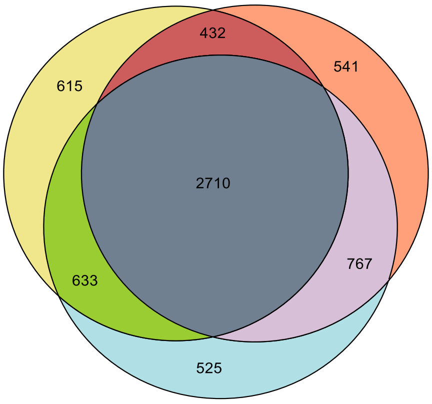

	Exclusive	Total	Label
A	615	4390	HCT116_5ul_CVSet1
B	541	4450	HCT116_5ul_CVSet2
C	525	4635	HCT116_5ul_CVSet3
A|B	432	3142	HCT116_5ul_CVSet1 | HCT116_5ul_CVSet2
B|C	767	3477	HCT116_5ul_CVSet2 | HCT116_5ul_CVSet3
A|C	633	3343	HCT116_5ul_CVSet1 | HCT116_5ul_CVSet3
A|B|C	2710	2710	HCT116_5ul_CVSet1 | HCT116_5ul_CVSet2 | HCT116_5ul_CVSet3
Sum	6223		

b)

A: CVSet1: by CV -40/-60/-80 V

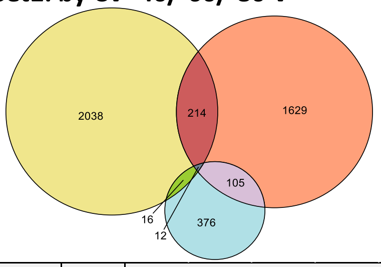

	Exclusive	Total	Label
A	2038	2280	Comp. Voltage [V]: -40
B	1629	1960	Comp. Voltage [V]: -60
C	375	509	Comp. Voltage [V]: -80
A|B	214	226	Comp. Voltage [V]: -40 | Comp. Voltage [V]: -60
B|C	105	117	Comp. Voltage [V]: -60 | Comp. Voltage [V]: -80
A|C	16	28	Comp. Voltage [V]: -40 | Comp. Voltage [V]: -80
A|B|C	12	12	Comp. Voltage [V]: -40 | Comp. Voltage [V]: -60 | Comp. Voltage [V]: -80
Sum	4390		

B: CVSet2: by CV -35/-50/-70 V

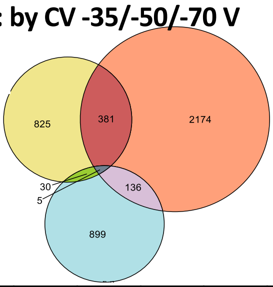

	Exclusive	Total	Label
A	85	1241	Comp. Voltage [V]: -35
B	2174	2696	Comp. Voltage [V]: -50
C	899	1070	Comp. Voltage [V]: -70
A|B	381	386	Comp. Voltage [V]: -40 | Comp. Voltage [V]: -60
B|C	136	141	Comp. Voltage [V]: -60 | Comp. Voltage [V]: -80
A|C	30	35	Comp. Voltage [V]: -40 | Comp. Voltage [V]: -80
A|B|C	5	5	Comp. Voltage [V]: -40 | Comp. Voltage [V]: -60 | Comp. Voltage [V]: -80
Sum	4450		

C: CVSet3: by CV -45/-55/-65 V

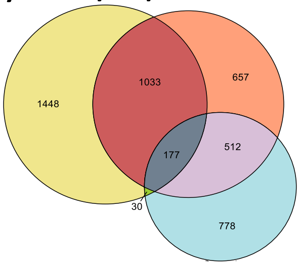

	Exclusive	Total	Label
A	1448	2688	Comp. Voltage [V]: -45
B	657	2379	Comp. Voltage [V]: -55
C	778	1497	Comp. Voltage [V]: -65
A|B	1033	1210	Comp. Voltage [V]: -40 | Comp. Voltage [V]: -60
B|C	512	689	Comp. Voltage [V]: -60 | Comp. Voltage [V]: -80
A|C	30	207	Comp. Voltage [V]: -40 | Comp. Voltage [V]: -80
A|B|C	177	177	Comp. Voltage [V]: -40 | Comp. Voltage [V]: -60 | Comp. Voltage [V]: -80
Sum	4635		

c)

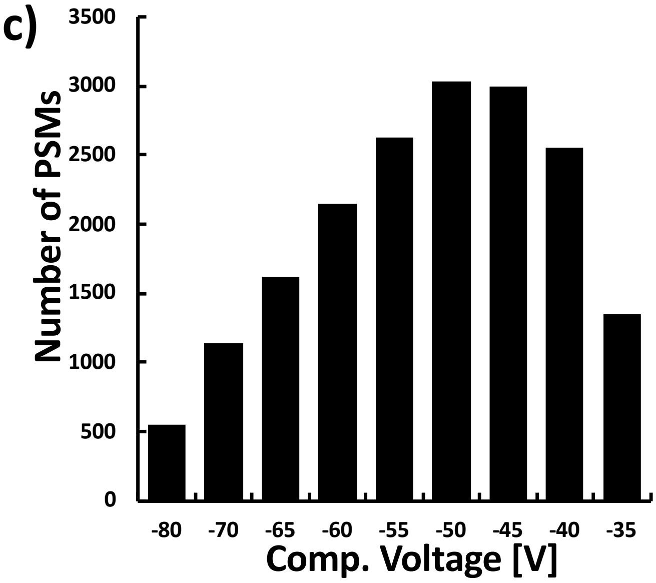

Supplementary Figure 3. Representative schematic diagram of global immunopeptidomics for HCT116 cells. a) The number of cells used, FAIMS-assisted analytical conditions, counts of MS/MS, PSMs, peptide groups, protein groups and the overlap of identified peptide groups in each CVset are depicted with a Venn diagram. **b)** The three Venn diagrams show the breakdown of the number of peptide groups belonging to each CV. **c)** A bar graph depicts the PSM counts across 9 CVs identified by global-immunopeptidomics.

Supplementary Figure 4

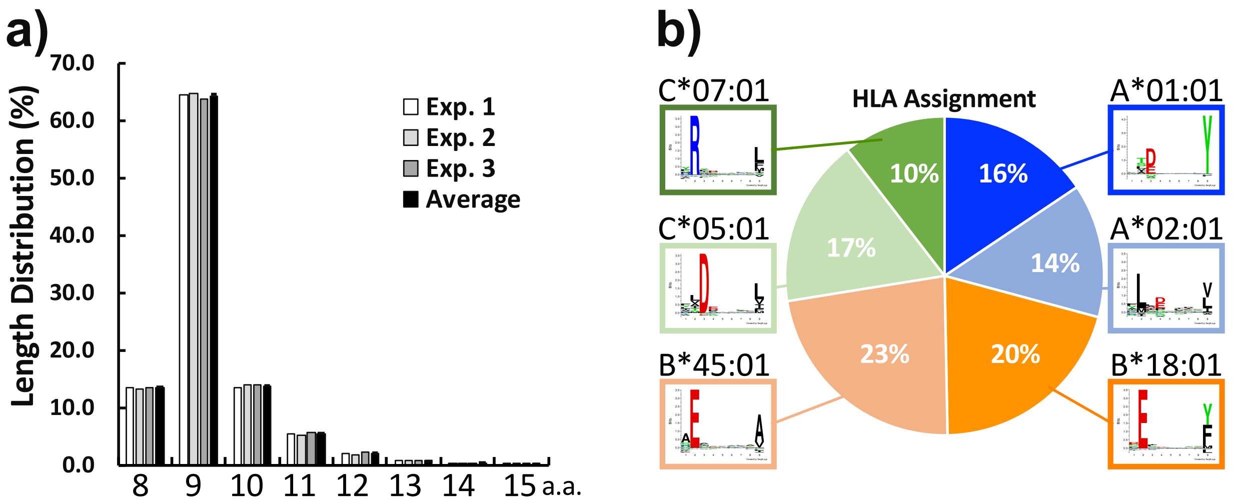

Supplementary Figure 4. Characteristics of the immunopeptidome of the colon cancer cell line HCT116. a) The length distribution of immunopeptides identified from HCT116 cells. The lengths of immunopeptides identified in Figure 1D were quantified. **b)** Representative image of unsupervised clustering of HLA motifs and the population of HLA assignments in the context of HLA allotypes.

Supplementary Figure5

a)

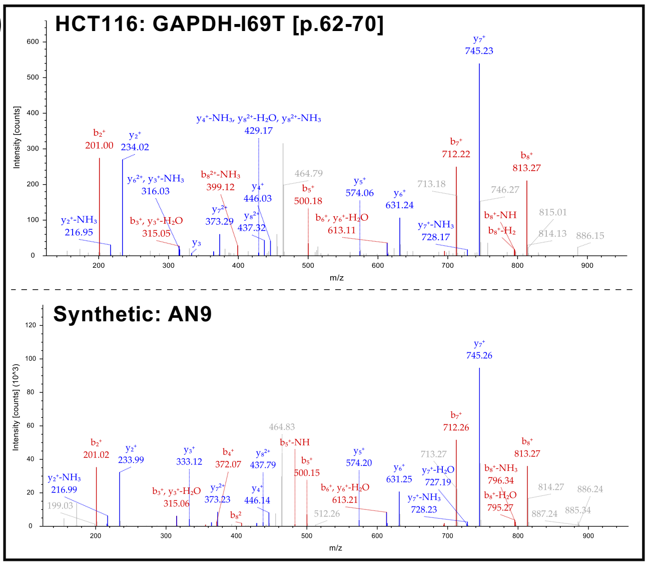

#1	b^+	b^{2+}	Seq.	y^+	y^{2+}	#2
1	72.04439	36.52583	A			9
2	201.08698	101.04713	E	874.46287	437.73507	8
3	315.12991	158.06859	N	745.42028	373.21378	7
4	372.15137	186.57933	G	631.37735	316.19231	6
5	500.24634	250.62681	K	574.35589	287.68158	5
6	613.33040	307.16884	L	446.26092	223.63410	4
7	712.39882	356.70305	V	333.17686	167.09207	3
8	813.44649	407.22689	T	234.10845	117.55786	2
9			N	133.06077	67.03402	1

Sequence: AENGKLVTN, Charge: +2, Monoisotopic m/z: 473.25333 Da (-0.31 mmu/-0.64 ppm), MH+: 945.49938 Da, RT: 17.6820 min,
Identified with: Sequest HT (v1.17); XCorr:2.00,
Fragment match tolerance used for search: 0.6 Da
Fragments used for search: $-H_2O$; y; $-NH_3$; y; b; b; $-H_2O$; b; $-NH_3$; y

#1	b^+	b^{2+}	Seq.	y^+	y^{2+}	#2
1	72.04439	36.52583	A			9
2	201.08698	101.04713	E	874.46287	437.73507	8
3	315.12991	158.06859	N	745.42028	373.21378	7
4	372.15137	186.57933	G	631.37735	316.19231	6
5	500.24634	250.62681	K	574.35589	287.68158	5
6	613.33040	307.16884	L	446.26092	223.63410	4
7	712.39882	356.70305	V	333.17686	167.09207	3
8	813.44649	407.22689	T	234.10845	117.55786	2
9			N	133.06077	67.03402	1

Sequence: AENGKLVTN, Charge: +2, Monoisotopic m/z: 473.25323 Da (-0.4 mmu/-0.84 ppm), MH+: 945.49919 Da, RT: 17.8667 min,
Identified with: Sequest HT (v1.17); XCorr:2.57,
Fragment match tolerance used for search: 0.6 Da
Fragments used for search: $-H_2O$; y; $-NH_3$; y; b; b; $-H_2O$; b; $-NH_3$; y

b)

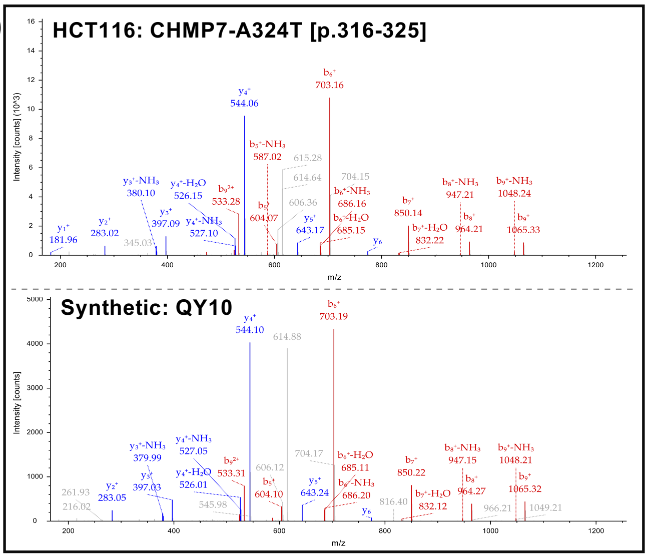

#1	b^+	b^{2+}	Seq.	y^+	y^{2+}	#2
1	129.06585	65.03657	Q			10
2	230.11353	115.56040	T	1118.48229	559.74478	9
3	345.14048	173.07388	D	1017.43461	509.22094	8
4	473.19905	237.10316	Q	902.40767	451.70747	7
5	604.23954	302.62341	M	774.34909	387.67818	6
6	703.30795	352.15761	V	643.30860	322.15794	5
7	850.37637	425.69182	F	544.24019	272.62373	4
8	964.41929	482.71328	N	397.17178	199.08953	3
9	1065.46697	533.23712	T	283.12885	142.06806	2
10			Y	182.08117	91.54422	1

Sequence: QTDQMVFNTY, Charge: +2, Monoisotopic m/z: 623.77393 Da (-0.14 mmu/-0.23 ppm), MH+: 1246.54057 Da, RT: 44.9058 min,
Identified with: Sequest HT (v1.17); XCorr:1.32,
Fragment match tolerance used for search: 0.6 Da
Fragments used for search: $-H_2O$; y; $-NH_3$; y; b; b; $-H_2O$; b; $-NH_3$; y

#1	b^+	b^{2+}	Seq.	y^+	y^{2+}	#2
1	129.06585	65.03657	Q			10
2	230.11353	115.56040	T	1118.48229	559.74478	9
3	345.14048	173.07388	D	1017.43461	509.22094	8
4	473.19905	237.10316	Q	902.40767	451.70747	7
5	604.23954	302.62341	M	774.34909	387.67818	6
6	703.30795	352.15761	V	643.30860	322.15794	5
7	850.37637	425.69182	F	544.24019	272.62373	4
8	964.41929	482.71328	N	397.17178	199.08953	3
9	1065.46697	533.23712	T	283.12885	142.06806	2
10			Y	182.08117	91.54422	1

Sequence: QTDQMVFNTY, Charge: +2, Monoisotopic m/z: 623.77405 Da (-0.02 mmu/-0.04 ppm), MH+: 1246.54082 Da, RT: 45.1203 min,
Identified with: Sequest HT (v1.17); XCorr:1.48,
Fragment match tolerance used for search: 0.6 Da
Fragments used for search: $-H_2O$; y; $-NH_3$; y; b; b; $-H_2O$; b; $-NH_3$; y

c)

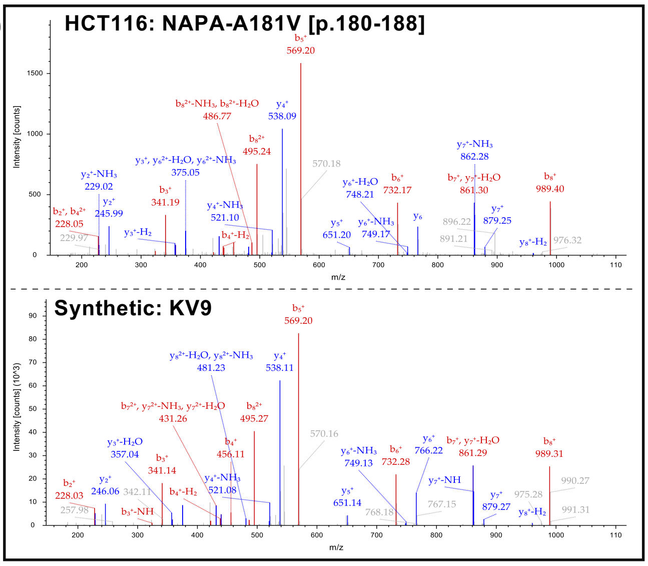

#1	b^+	b^{2+}	Seq.	y^+	y^{2+}	#2
1	129.10224	65.05476	K			9
2	228.17065	114.58897	V	978.51424	489.76076	8
3	341.25472	171.13100	I	879.44582	440.22655	7
4	456.28166	228.64447	D	766.36176	383.68452	6
5	569.36572	285.18650	I	651.33482	326.17105	5
6	732.42905	366.71816	Y	538.25075	269.62902	4
7	861.47165	431.23946	E	375.18743	188.09735	3
8	989.53022	495.26875	Q	246.14483	123.57605	2
9			V	118.08626	59.54677	1

Sequence: KVIDIYEQV, Charge: +2, Monoisotopic m/z: 553.80896 Da (+0.72 mmu/+1.3 ppm), MH+: 1106.61064 Da, RT: 39.2498 min,
Identified with: Sequest HT (v1.17); XCorr:2.63,
Fragment match tolerance used for search: 0.6 Da
Fragments used for search: $-H_2O$; y; $-NH_3$; y; b; b; $-H_2O$; b; $-NH_3$; y

#1	b^+	b^{2+}	Seq.	y^+	y^{2+}	#2
1	129.10224	65.05476	K			9
2	228.17065	114.58897	V	978.51424	489.76076	8
3	341.25472	171.13100	I	879.44582	440.22655	7
4	456.28166	228.64447	D	766.36176	383.68452	6
5	569.36572	285.18650	I	651.33482	326.17105	5
6	732.42905	366.71816	Y	538.25075	269.62902	4
7	861.47165	431.23946	E	375.18743	188.09735	3
8	989.53022	495.26875	Q	246.14483	123.57605	2
9			V	118.08626	59.54677	1

Sequence: KVIDIYEQV, Charge: +2, Monoisotopic m/z: 553.80841 Da (+0.17 mmu/+0.31 ppm), MH+: 1106.60954 Da, RT: 39.1698 min,
Identified with: Sequest HT (v1.17); XCorr:2.72,
Fragment match tolerance used for search: 0.6 Da
Fragments used for search: $-H_2O$; y; $-NH_3$; y; b; b; $-H_2O$; b; $-NH_3$; y

Supplementary Figure 5. Spectral comparison of representative neoantigens identified from HCT116 cells by cognate synthetic peptides. The MS2 spectra obtained from an on-column volume of 1 µl (5e6 cells) of GAPDH-I69T (a), an on-column volume of 4 µl (2e7 cells) of CHMP7-A324T (b) and an on-column volume of 1 µl (5e6 cells) of NAPA-A181V (c) by global-immunopeptidomics are shown. Gray peaks are the background peaks that are provably derived from the irrelevant precursor ions in the same MS2 scan window.

Supplementary Figure 6

a)

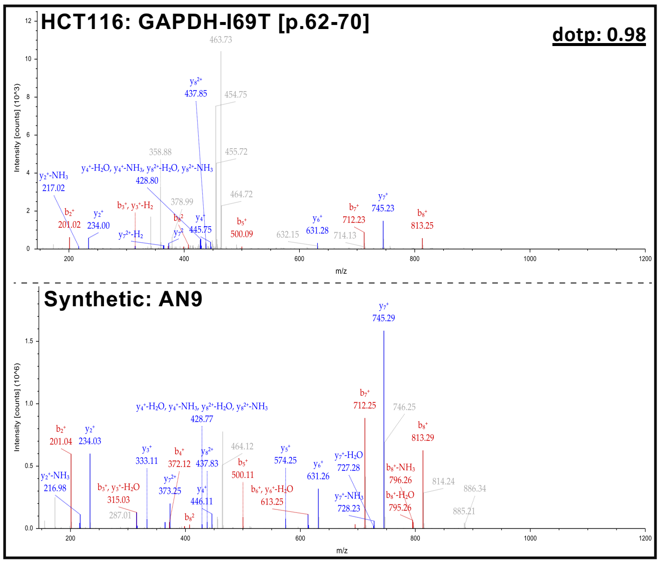

#1	b^+	b^{2+}	Seq.	y^+	y^{2+}	#2
1	72.04439	36.52583	A			9
2	201.08698	101.04713	E	874.46287	437.73507	8
3	315.12991	158.06859	N	745.42028	373.21378	7
4	372.15137	186.57933	G	631.37735	316.19231	6
5	500.24634	250.62681	K	574.35589	287.68158	5
6	613.33040	307.16884	L	446.26092	223.63410	4
7	712.39882	356.70305	V	333.17686	167.09207	3
8	813.44649	407.22689	T	234.10845	117.55786	2
9			N	133.06077	67.03402	1

Sequence: AENGKLVTN, Charge: +2, Monoisotopic m/z: 473.25360 Da (-0.03 mmu/-0.06 ppm), MH+: 945.49993 Da, RT: 17.6474 min,
Identified with: Sequest HT (v1.17); XCorr:1.82,
Fragment match tolerance used for search: 0.6 Da
Fragments used for search: $-H_2O$; y; $-NH_3$; y; b; b; $-H_2O$; b; $-NH_3$; y

#1	b^+	b^{2+}	Seq.	y^+	y^{2+}	#2
1	72.04439	36.52583	A			9
2	201.08698	101.04713	E	874.46287	437.73507	8
3	315.12991	158.06859	N	745.42028	373.21378	7
4	372.15137	186.57933	G	631.37735	316.19231	6
5	500.24634	250.62681	K	574.35589	287.68158	5
6	613.33040	307.16884	L	446.26092	223.63410	4
7	712.39882	356.70305	V	333.17686	167.09207	3
8	813.44649	407.22689	T	234.10845	117.55786	2
9			N	133.06077	67.03402	1

Sequence: AENGKLVTN, Charge: +2, Monoisotopic m/z: 473.25360 Da (-0.03 mmu/-0.06 ppm), MH+: 945.49993 Da, RT: 17.6551 min,
Identified with: Sequest HT (v1.17); XCorr:3.02,
Fragment match tolerance used for search: 0.6 Da
Fragments used for search: $-H_2O$; y; $-NH_3$; y; b; b; $-H_2O$; b; $-NH_3$; y

b)

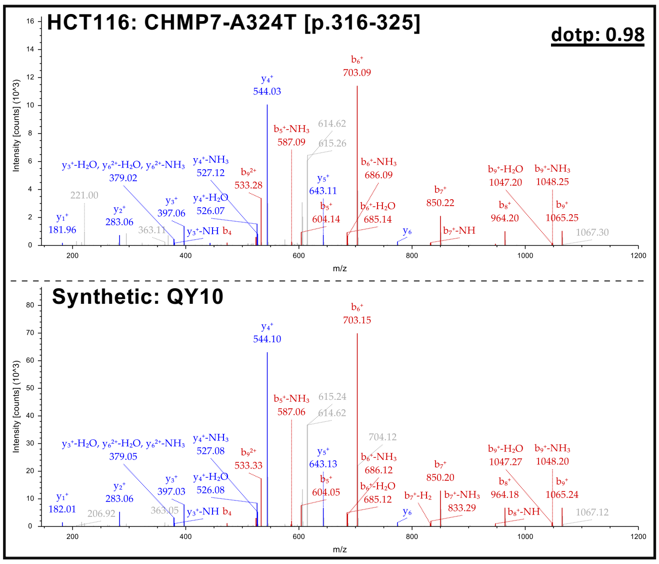

#1	b^+	b^{2+}	Seq.	y^+	y^{2+}	#2
1	129.06585	65.03657	Q			10
2	230.11353	115.56040	T	1118.48229	559.74478	9
3	345.14048	173.07388	D	1017.43461	509.22094	8
4	473.19905	237.10316	Q	902.40767	451.70747	7
5	604.23954	302.62341	M	774.34909	387.67818	6
6	703.30795	352.15761	V	643.30860	322.15794	5
7	850.37637	425.69182	F	544.24019	272.62373	4
8	964.41929	482.71328	N	397.17178	199.08953	3
9	1065.46697	533.23712	T	283.12885	142.06806	2
10			Y	182.08117	91.54422	1

Sequence: QTDQMVFNTY, Charge: +2, Monoisotopic m/z: 623.77411 Da (+0.04 mmu/+0.06 ppm), MH+: 1246.54094 Da, RT: 44.8999 min,
Identified with: Sequest HT (v1.17); XCorr:1.41,
Fragment match tolerance used for search: 0.6 Da
Fragments used for search: $-H_2O$; y; $-NH_3$; y; b; b; $-H_2O$; b; $-NH_3$; y

#1	b^+	b^{2+}	Seq.	y^+	y^{2+}	#2
1	129.06585	65.03657	Q			10
2	230.11353	115.56040	T	1118.48229	559.74478	9
3	345.14048	173.07388	D	1017.43461	509.22094	8
4	473.19905	237.10316	Q	902.40767	451.70747	7
5	604.23954	302.62341	M	774.34909	387.67818	6
6	703.30795	352.15761	V	643.30860	322.15794	5
7	850.37637	425.69182	F	544.24019	272.62373	4
8	964.41929	482.71328	N	397.17178	199.08953	3
9	1065.46697	533.23712	T	283.12885	142.06806	2
10			Y	182.08117	91.54422	1

Sequence: QTDQMVFNTY, Charge: +2, Monoisotopic m/z: 623.77411 Da (+0.04 mmu/+0.06 ppm), MH+: 1246.54094 Da, RT: 44.8462 min,
Identified with: Sequest HT (v1.17); XCorr:1.64,
Fragment match tolerance used for search: 0.6 Da
Fragments used for search: $-H_2O$; y; $-NH_3$; y; b; b; $-H_2O$; b; $-NH_3$; y

c)

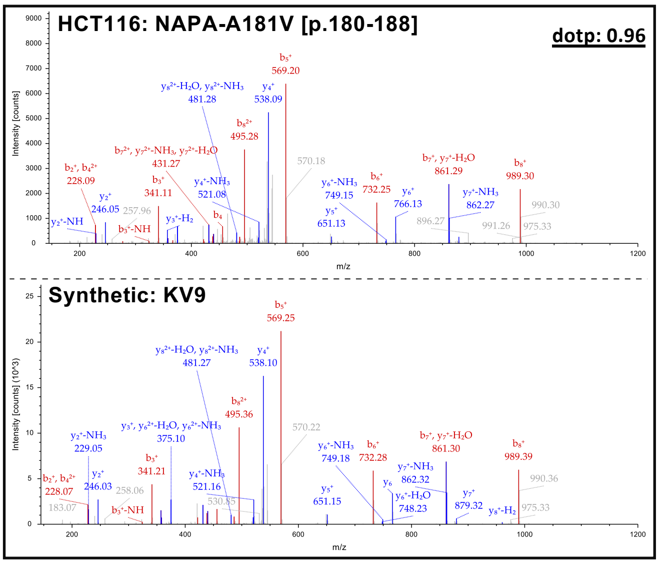

#1	b^+	b^{2+}	Seq.	y^+	y^{2+}	#2
1	129.10224	65.05476	K			9
2	228.17065	114.58897	V	978.51424	489.76076	8
3	341.25472	171.13100	I	879.44582	440.22655	7
4	456.28166	228.64447	D	766.36176	383.68452	6
5	569.36572	285.18650	I	651.33482	326.17105	5
6	732.42905	366.71816	Y	538.25075	269.62902	4
7	861.47165	431.23946	E	375.18743	188.09735	3
8	989.53022	495.26875	Q	246.14483	123.57605	2
9			V	118.08626	59.54677	1

Sequence: KVIDIYEQV, Charge: +2, Monoisotopic m/z: 553.80823 Da (-0.01 mmu/-0.02 ppm), MH+: 1106.60918 Da, RT: 39.2272 min,
Identified with: Sequest HT (v1.17); XCorr:2.52,
Fragment match tolerance used for search: 0.6 Da
Fragments used for search: $-H_2O$; y; $-NH_3$; y; b; b; $-H_2O$; b; $-NH_3$; y

#1	b^+	b^{2+}	Seq.	y^+	y^{2+}	#2
1	129.10224	65.05476	K			9
2	228.17065	114.58897	V	978.51424	489.76076	8
3	341.25472	171.13100	I	879.44582	440.22655	7
4	456.28166	228.64447	D	766.36176	383.68452	6
5	569.36572	285.18650	I	651.33482	326.17105	5
6	732.42905	366.71816	Y	538.25075	269.62902	4
7	861.47165	431.23946	E	375.18743	188.09735	3
8	989.53022	495.26875	Q	246.14483	123.57605	2
9			V	118.08626	59.54677	1

Sequence: KVIDIYEQV, Charge: +2, Monoisotopic m/z: 553.80823 Da (-0.01 mmu/-0.02 ppm), MH+: 1106.60918 Da, RT: 39.1608 min,
Identified with: Sequest HT (v1.17); XCorr:2.92,
Fragment match tolerance used for search: 0.6 Da
Fragments used for search: $-H_2O$; y; $-NH_3$; y; b; b; $-H_2O$; b; $-NH_3$; y

Supplementary Figure 6. Spectral comparison of representative neoantigens identified from HCT116 cells by cognate synthetic peptides. The MS2 spectra of GAPDH-I69T **(a)**, CHMP7-A324T **(b)** and NAPA-A181V **(c)** produced by targeted-immunopeptidomics are shown. All MS2 spectra shown in this figure were obtained from an on-column volume of 1 µl (5e6 cells). To verify the similarity of MS2 spectra more objectively, we introduced Skyline software to calculate the dot-product (dotp) score under the same data acquisition settings and confirmed the good correlation score (> 0.95) from each analysis. Gray peaks are the background noise, which is provably derived from irrelevant precursor ions for identification.

Supplementary Figure 7

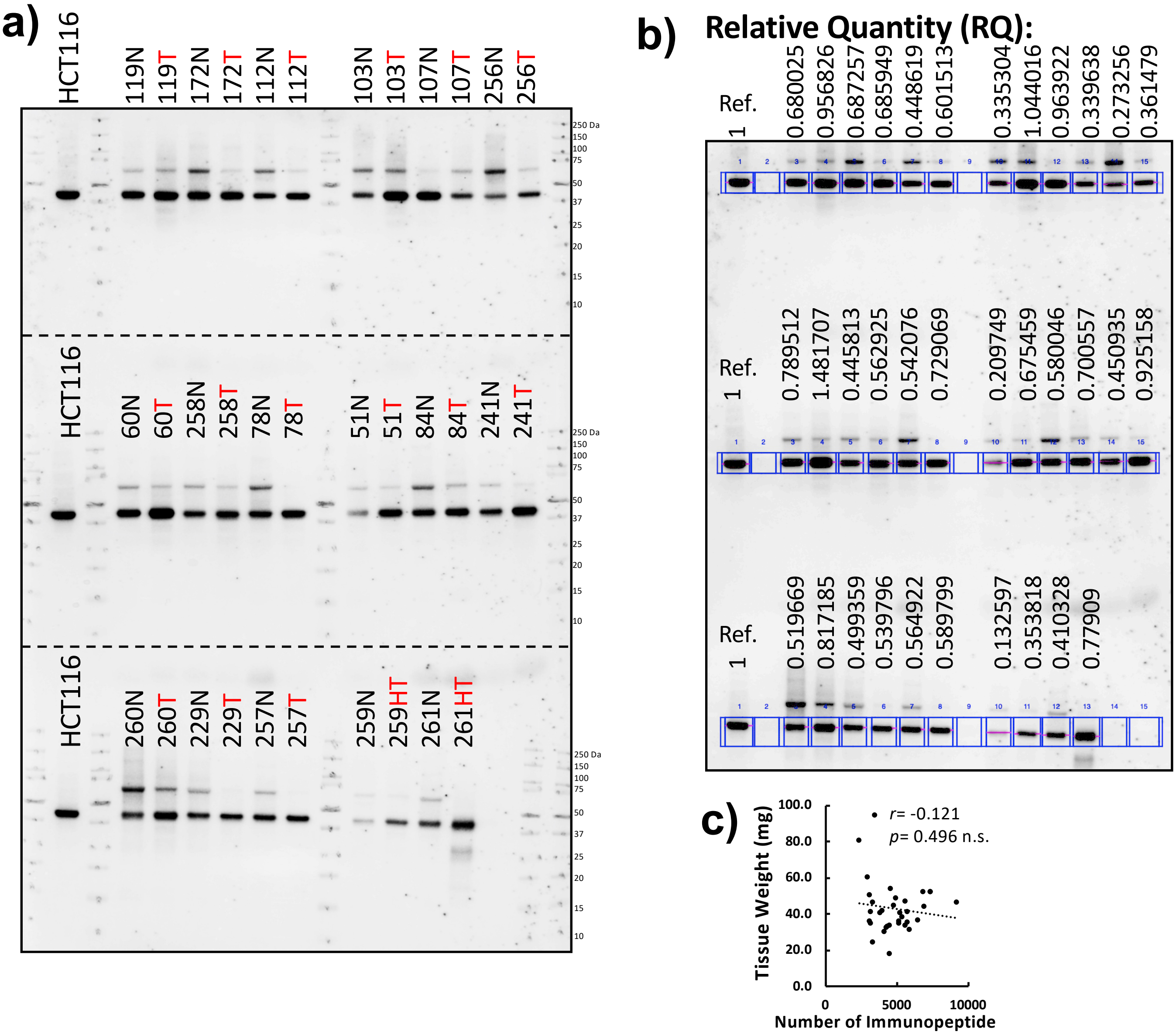

Supplementary Figure 7. Expression and quantification of HLA in tissue samples. a) Western blot of HLA class I expression was assessed by anti-alpha-chain antibody (Clone: EMR8-5). Identical HCT116 lysate was loaded onto each gel as a detection control. **b).** The relative quantity of alpha-chain was calculated by Image Lab software. The HCT116 bands were used as a reference control (relative quantity (RQ)= 1). The calculated RQs are shown numerically. **c)** Unlike the protein amount and relative quantity of the α-chain (shown in Figure 2g and 2 h), there was no statistically significant correlation between the number of immunopeptides identified and the tissue weight in the samples.

Supplementary Figure 8

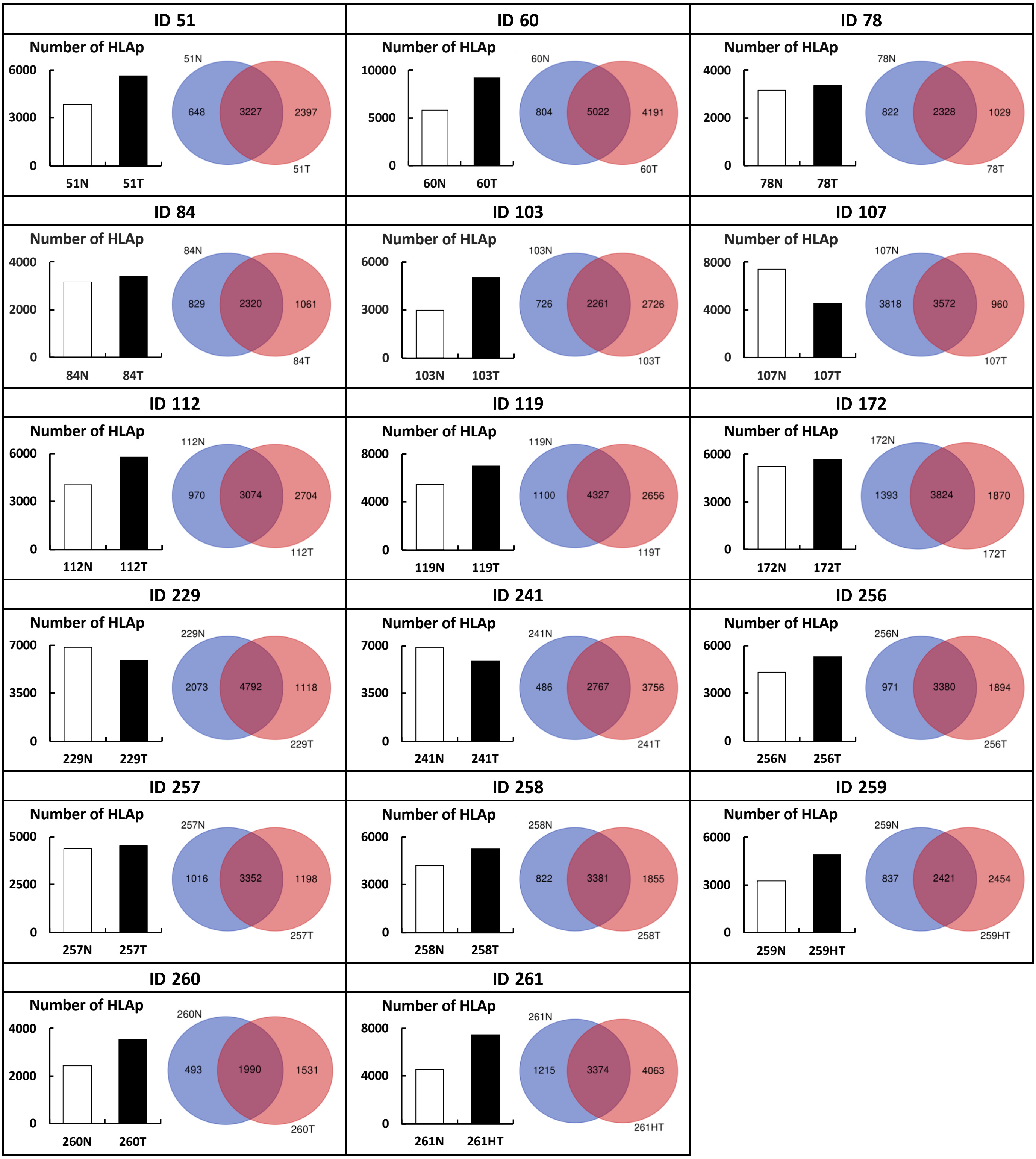

Supplementary Figure 8. Number of immunopeptides per sample per patient. The panel shows the details of the identified number of immunopeptides in each normal and tumor tissue sample, and the overlap of immunopeptides between normal and tumor was calculated by a Venn diagram per person. The Venn diagram does not reflect the sample size in this figure.

Supplementary Figure 9

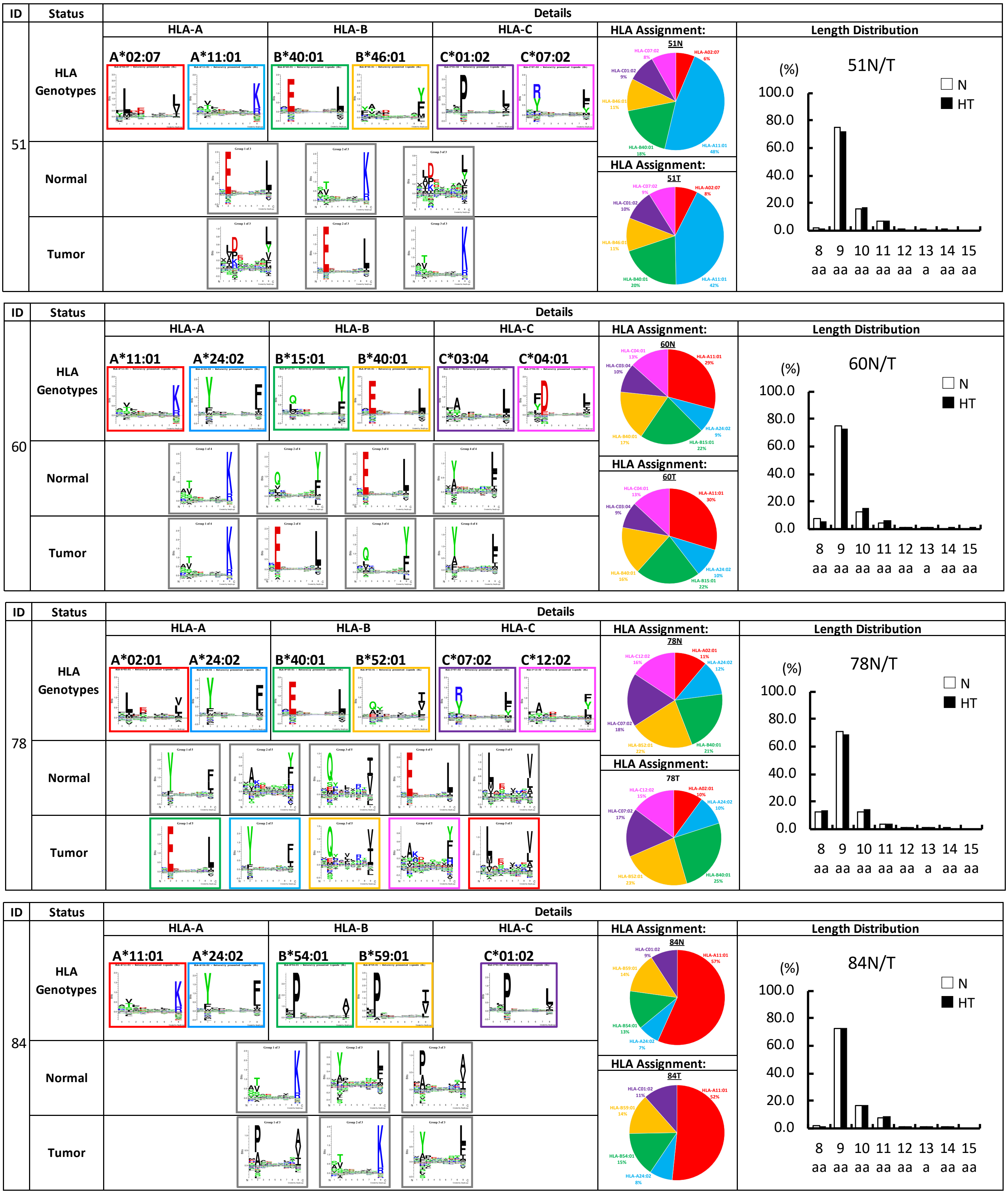

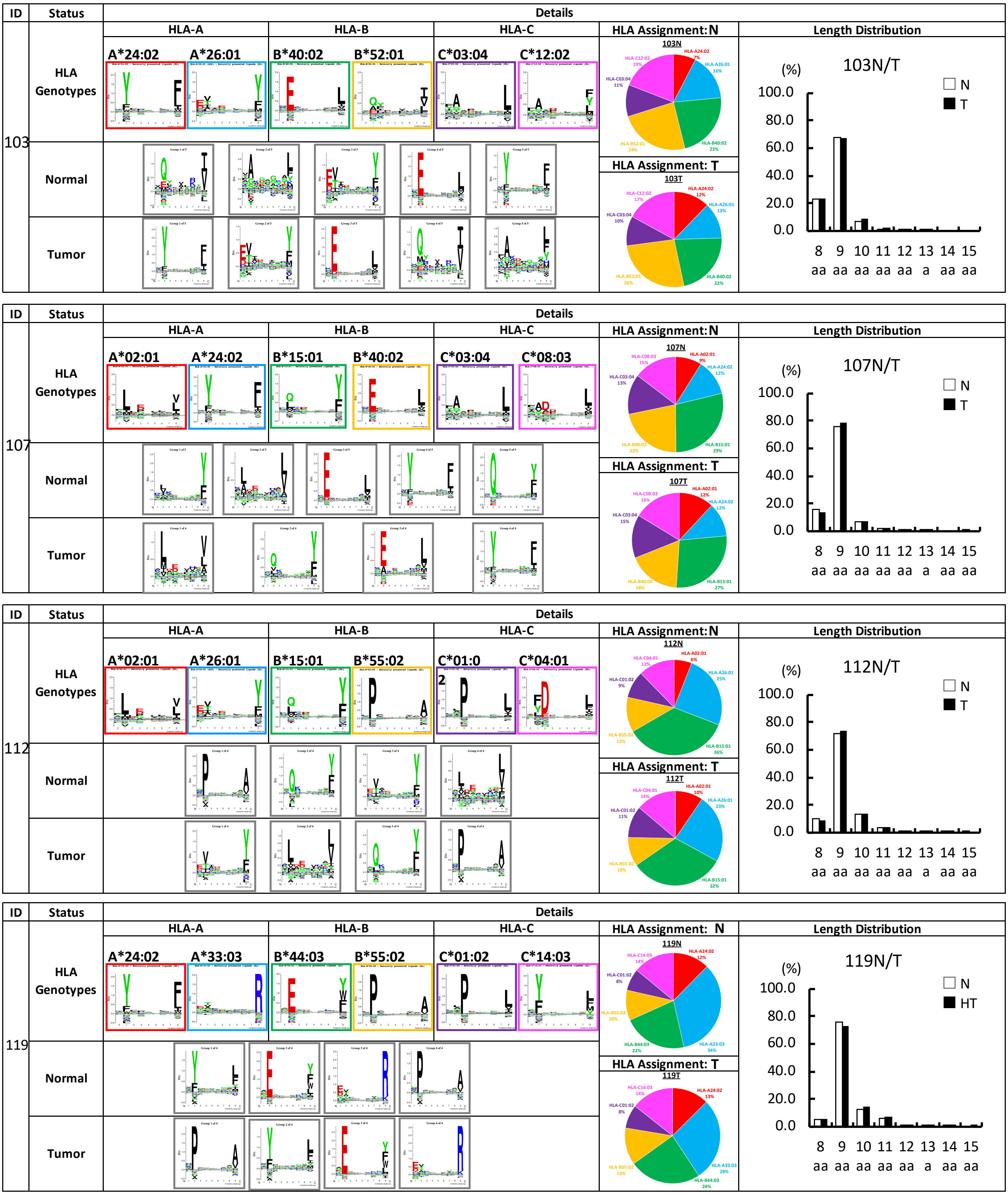

ID
Status
Details
HLA-A
HLA-B
HLA-C
HLA Assignment: N
HLA Assignment: T
Length Distribution
103
HLA Genotypes
A*24:02
A*26:01
B*40:02
B*52:01
C*03:04
C*12:02
Normal
Tumor
103N
103T
103N/T
(%)
100.0
80.0
60.0
40.0
20.0
0.0
N
T
8 aa
9 aa
10 aa
11 aa
12 aa
13 a
14 aa
15 aa
107
A*02:01
A*24:02
B*15:01
B*40:02
C*03:04
C*08:03
107N
107T
107N/T
112
A*02:01
A*26:01
B*15:01
B*55:02
C*01:0 2
C*04:01
112N
112T
112N/T
119
A*24:02
A*33:03
B*44:03
B*55:02
C*01:02
C*14:03
119N
119T
119N/T
HT

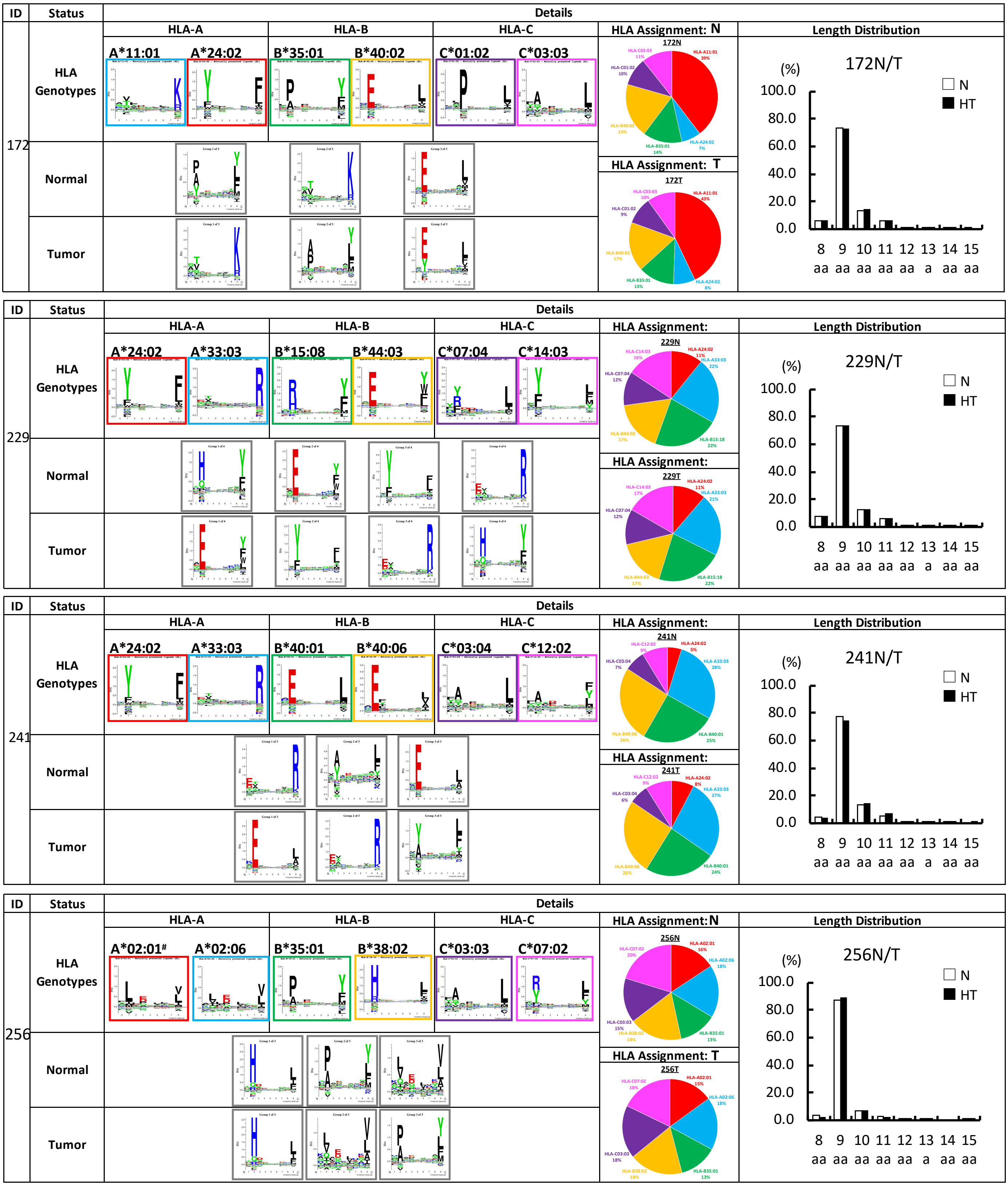

ID
Status
Details
172
HLA Genotypes
HLA-A
A*11:01
A*24:02
HLA-B
B*35:01
B*40:02
HLA-C
C*01:02
C*03:03
HLA Assignment: N
172N
HLA-A11:01 39%
HLA-A24:02 7%
HLA-B35:01 14%
HLA-B40:02 19%
HLA-C01:02 10%
HLA-C03:03 11%
HLA Assignment: T
172T
HLA-A11:01 43%
HLA-A24:02 8%
HLA-B35:01 13%
HLA-B40:02 17%
HLA-C01:02 9%
HLA-C03:03 10%
Normal
Tumor
Length Distribution
172N/T
(%)
100.0
80.0
60.0
40.0
20.0
0.0
N
HT
8 aa
9 aa
10 aa
11 aa
12 aa
13 a
14 aa
15 aa
229
HLA-A
A*24:02
A*33:03
HLA-B
B*15:08
B*44:03
HLA-C
C*07:04
C*14:03
HLA Assignment:
229N
HLA-A24:02 11%
HLA-A33:03 22%
HLA-B15:18 22%
HLA-B44:03 17%
HLA-C07:04 12%
HLA-C14:03 16%
229T
HLA-A24:02 11%
HLA-A33:03 21%
HLA-B15:18 22%
HLA-B44:03 17%
HLA-C07:04 12%
HLA-C14:03 17%
229N/T
241
HLA-A
A*24:02
A*33:03
HLA-B
B*40:01
B*40:06
HLA-C
C*03:04
C*12:02
241N
HLA-A24:02 5%
HLA-A33:03 28%
HLA-B40:01 25%
HLA-B40:06 26%
HLA-C03:04 7%
HLA-C12:02 9%
241T
HLA-A24:02 8%
HLA-A33:03 27%
HLA-B40:01 24%
HLA-B40:06 26%
HLA-C03:04 6%
HLA-C12:02 9%
241N/T
256
HLA-A
A*02:01#
A*02:06
HLA-B
B*35:01
B*38:02
HLA-C
C*03:03
C*07:02
HLA Assignment: N
256N
HLA-A02:01 16%
HLA-A02:06 18%
HLA-B35:01 13%
HLA-B38:02 18%
HLA-C03:03 15%
HLA-C07:02 20%
HLA Assignment: T
256T
HLA-A02:01 15%
HLA-A02:06 18%
HLA-B35:01 13%
HLA-B38:02 18%
HLA-C03:03 18%
HLA-C07:02 18%
256N/T

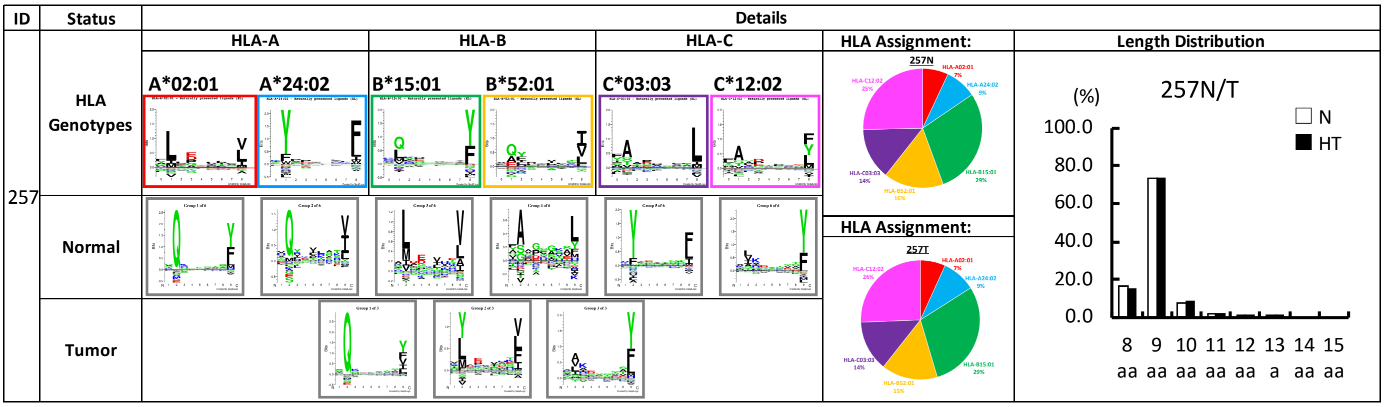
ID
Status
Details
HLA-A
HLA-B
HLA-C
HLA Assignment:
Length Distribution
257
HLA Genotypes
A*02:01
A*24:02
B*15:01
B*52:01
C*03:03
C*12:02
Normal
Tumor
257N
HLA-A02:01 7%
HLA-A24:02 9%
HLA-B15:01 29%
HLA-B52:01 16%
HLA-C03:03 14%
HLA-C12:02 25%
257T
HLA-A02:01 7%
HLA-A24:02 9%
HLA-B15:01 29%
HLA-B52:01 15%
HLA-C03:03 14%
HLA-C12:02 26%
(%)
257N/T
N
HT
100.0
80.0
60.0
40.0
20.0
0.0
8 aa
9 aa
10 aa
11 aa
12 aa
13 a
14 aa
15 aa

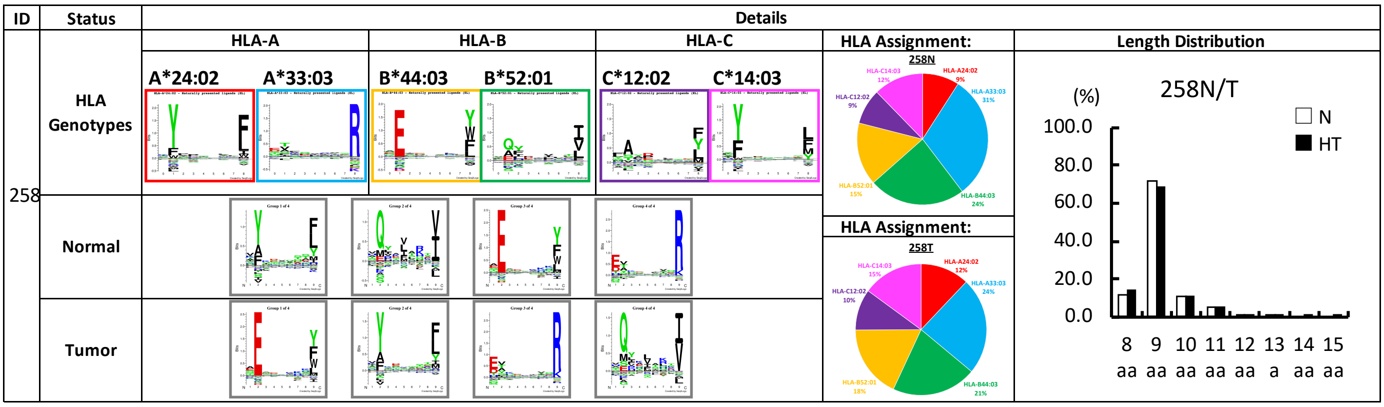
ID
Status
Details
HLA-A
HLA-B
HLA-C
HLA Assignment:
Length Distribution
258
HLA Genotypes
A*24:02
A*33:03
B*44:03
B*52:01
C*12:02
C*14:03
Normal
Tumor
258N
HLA-A24:02 9%
HLA-A33:03 31%
HLA-B44:03 24%
HLA-B52:01 15%
HLA-C12:02 9%
HLA-C14:03 12%
258T
HLA-A24:02 12%
HLA-A33:03 24%
HLA-B44:03 21%
HLA-B52:01 18%
HLA-C12:02 10%
HLA-C14:03 15%
(%)
258N/T
N
HT
100.0
80.0
60.0
40.0
20.0
0.0
8 aa
9 aa
10 aa
11 aa
12 aa
13 a
14 aa
15 aa

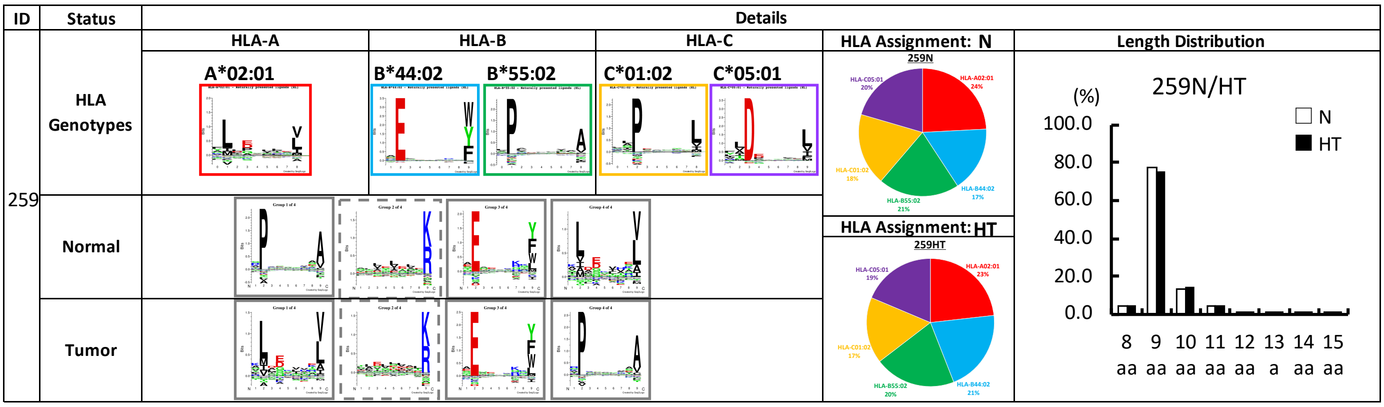
ID
Status
Details
HLA-A
HLA-B
HLA-C
HLA Assignment: N
HLA Assignment: HT
Length Distribution
259
HLA Genotypes
A*02:01
B*44:02
B*55:02
C*01:02
C*05:01
Normal
Tumor
259N
HLA-A02:01 24%
HLA-B44:02 17%
HLA-B55:02 21%
HLA-C01:02 18%
HLA-C05:01 20%
259HT
HLA-A02:01 23%
HLA-B44:02 21%
HLA-B55:02 20%
HLA-C01:02 17%
HLA-C05:01 19%
(%)
259N/HT
N
HT
100.0
80.0
60.0
40.0
20.0
0.0
8 aa
9 aa
10 aa
11 aa
12 aa
13 a
14 aa
15 aa

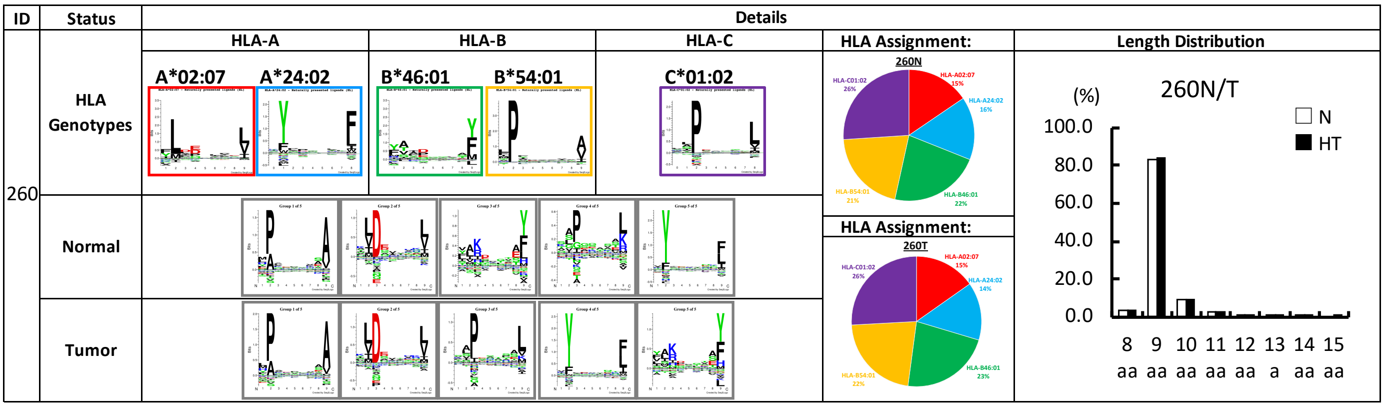
ID
Status
Details
HLA-A
HLA-B
HLA-C
HLA Assignment:
Length Distribution
260
HLA Genotypes
A*02:07
A*24:02
B*46:01
B*54:01
C*01:02
Normal
Tumor
260N
HLA-A02:07 15%
HLA-A24:02 16%
HLA-B46:01 22%
HLA-B54:01 21%
HLA-C01:02 26%
260T
HLA-A02:07 15%
HLA-A24:02 14%
HLA-B46:01 23%
HLA-B54:01 22%
HLA-C01:02 26%
(%)
260N/T
N
HT
100.0
80.0
60.0
40.0
20.0
0.0
8 aa
9 aa
10 aa
11 aa
12 aa
13 a
14 aa
15 aa

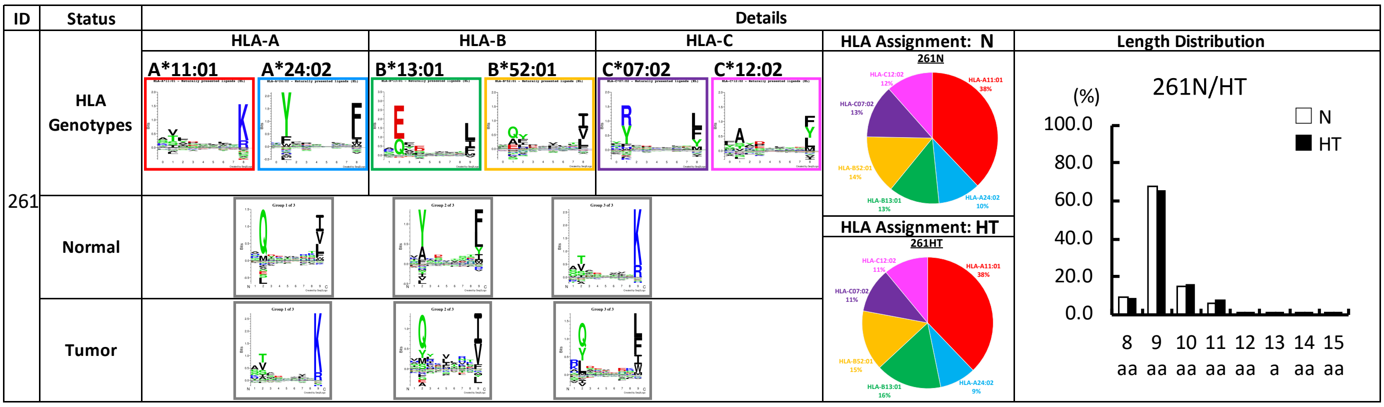

Supplementary Figure 9. Comparison of unsupervised clustering, assignment and the length of HLAps in the individual immunopeptidome. The HLA allotype and its motif sequence, unsupervised clustering of actually identified immunopeptides, immunopeptide assignment to corresponding HLA allotypes, and the length distribution of immunopeptides are shown individually. The length of immunopeptides was compared between normal and tumor tissues in identical patients. Among these analyzed parameters, there were no differences between the normal and tumor immunopeptidome in each patient.

Supplementary Figure 10

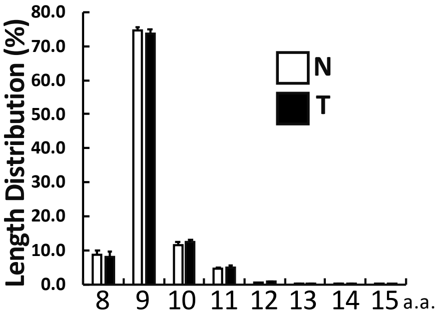

Supplementary Figure 10. Comparison of personalized immunopeptidome analysis for CRC tissue samples. **a)** The length distribution of immunopeptides (44,815 HLAps in total, as shown in Figure 2i). Peptides of nine amino acids in length dominated more than 75% of the overall immunopeptidome. Peptides eight to 12 amino acids in length covered more than 99.7% of the overall immunopeptidome in this study.

Supplementary Figure 11

a) **Colo668**

Sample preparation from 1e8 Colo668 Cells
Sample dissolved in 20ul of Injection Buffer
MS on-column volume: 6ul (≒ 3.0e7 cells)/ CVSet)
Total: 9.0e7 cells
Analyses (3 CVSets) → Total: 9 FAIMS CVs

	Number
MS/MS	73033
PSMs	27278
Peptide Groups	10474
Protein Groups	4956

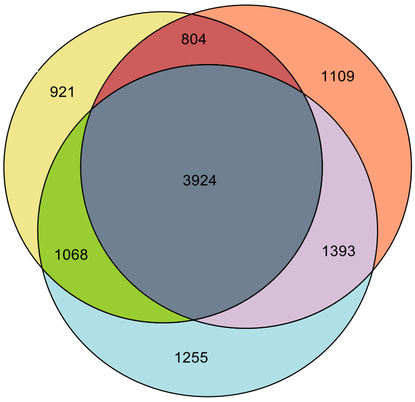

	Exclusive	Total	Label
A	921	6717	Colo668_6ul_CVSet1
B	1109	7230	Colo668_6ul_CVSet2
C	1255	7640	Colo668_6ul_CVSet3
A|B	804	4728	Colo668_6ul_CVSet1 | Colo668_6ul_CVSet2
B|C	1393	5317	Colo668_6ul_CVSet2 | Colo668_6ul_CVSet3
A|C	1068	4992	Colo668_6ul_CVSet1 | Colo668_6ul_CVSet3
A|B|C	3924	3924	Colo668_6ul_CVSet1 | Colo668_6ul_CVSet2 | Colo668_6ul_CVSet3
Sum	10474		

b)

A: CVSet1: by CV -40/-60/-80 V

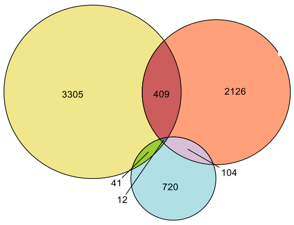

	Exclusive	Total	Label
A	3305	3767	Comp. Voltage [V]: -40
B	2126	2651	Comp. Voltage [V]: -60
C	720	877	Comp. Voltage [V]: -80
A|B	409	421	Comp. Voltage [V]: -40 | Comp. Voltage [V]: -60
B|C	104	116	Comp. Voltage [V]: -60 | Comp. Voltage [V]: -80
A|C	41	53	Comp. Voltage [V]: -40 | Comp. Voltage [V]: -80
A|B|C	12	12	Comp. Voltage [V]: -40 | Comp. Voltage [V]: -60 | Comp. Voltage [V]: -80
Sum	6717		

B: CVSet2: by CV -35/-50/-70 V

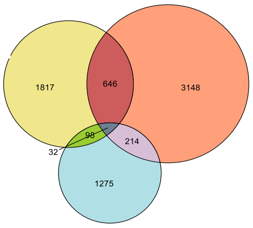

		Total	Label
A	1817	2593	Comp. Voltage [V]: -35
B	3148	4040	Comp. Voltage [V]: -50
C	1275	1619	Comp. Voltage [V]: -70
A|B	646	678	Comp. Voltage [V]: -40 | Comp. Voltage [V]: -60
B|C	214	246	Comp. Voltage [V]: -60 | Comp. Voltage [V]: -80
A|C	98	130	Comp. Voltage [V]: -40 | Comp. Voltage [V]: -80
A|B|C	32	32	Comp. Voltage [V]: -40 | Comp. Voltage [V]: -60 | Comp. Voltage [V]: -80
Sum	7230		

C: CVSet3: by CV -45/-55/-65 V

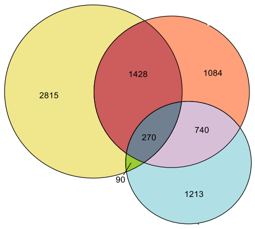

	Exclusive	Total	Label
A	2815	4603	Comp. Voltage [V]: -45
B	1084	3522	Comp. Voltage [V]: -55
C	1213	231	Comp. Voltage [V]: -65
A|B	1428	1698	Comp. Voltage [V]: -40 | Comp. Voltage [V]: -60
B|C	740	1010	Comp. Voltage [V]: -60 | Comp. Voltage [V]: -80
A|C	90	360	Comp. Voltage [V]: -40 | Comp. Voltage [V]: -80
A|B|C	270	270	Comp. Voltage [V]: -40 | Comp. Voltage [V]: -60 | Comp. Voltage [V]: -80
Sum	7640		

c)

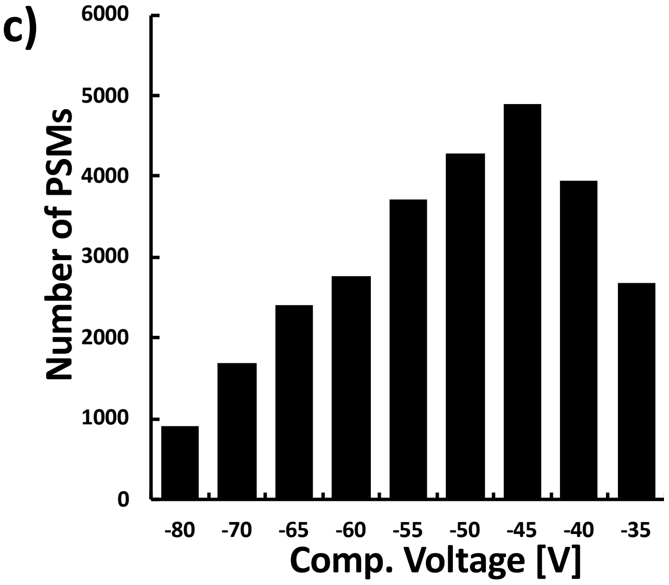

Supplementary Figure 11. Representative schematic diagram of global immunopeptidomics for Colo668. a) The number of cells used, FAIMS-assisted analytical conditions, counts of MS/MS, PSMs, peptide groups, protein groups and the overlap of identified peptide groups in each CVset with a Venn diagram are depicted. **b)** The three Venn diagrams show the breakdown of the number of peptide groups belonging to each CV. **c)** A bar graph depicts the PSM counts identified in each CV used in global-immunopeptidomics.

Supplementary Figure 12

a)

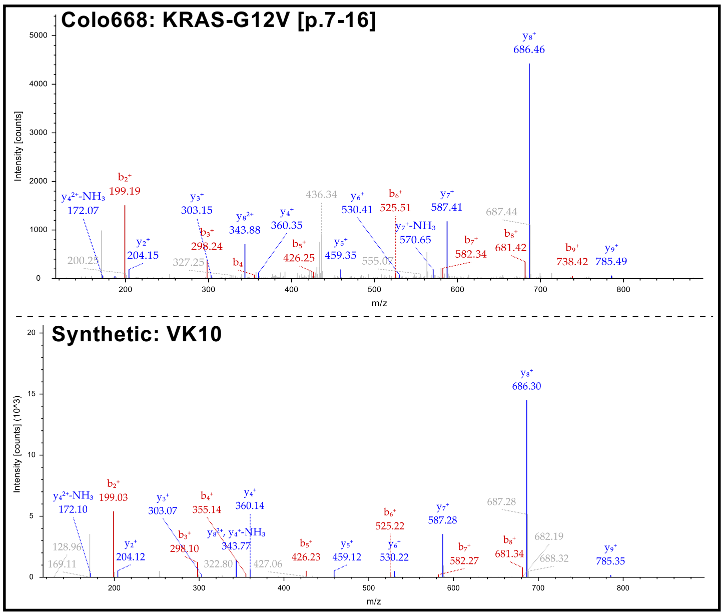

#1	b⁺	b²⁺	Seq.	y⁺	y²⁺	#2
1	100.07569	50.54148	V			10
2	199.14410	100.07569	V	785.48796	393.24762	9
3	298.21252	149.60990	V	686.41955	343.71341	8
4	355.23398	178.12063	G	587.35114	294.17921	7
5	426.27110	213.63919	A	530.32967	265.66847	6
6	525.33951	263.17339	V	459.29256	230.14992	5
7	582.36097	291.68412	G	360.22415	180.61571	4
8	681.42939	341.21833	V	303.20268	152.10498	3
9	738.45085	369.72906	G	204.13427	102.57077	2
10			K	147.11280	74.06004	1

Sequence: VVVGAVGVGK, Charge: +2, Monoisotopic m/z: 442.78186 Da (+0.03 mmu/+0.07 ppm), MH+: 884.55644 Da, RT: 26.7455 min,
Identified with: Sequest HT (v1.17); XCorr:2.30,
Fragment match tolerance used for search: 0.6 Da
Fragments used for search: -NH_3; y; b; y

#1	b⁺	b²⁺	Seq.	y⁺	y²⁺	#2
1	100.07569	50.54148	V			10
2	199.14410	100.07569	V	785.48796	393.24762	9
3	298.21252	149.60990	V	686.41955	343.71341	8
4	355.23398	178.12063	G	587.35114	294.17921	7
5	426.27110	213.63919	A	530.32967	265.66847	6
6	525.33951	263.17339	V	459.29256	230.14992	5
7	582.36097	291.68412	G	360.22415	180.61571	4
8	681.42939	341.21833	V	303.20268	152.10498	3
9	738.45085	369.72906	G	204.13427	102.57077	2
10			K	147.11280	74.06004	1

Sequence: VVVGAVGVGK, Charge: +2, Monoisotopic m/z: 442.78171 Da (-0.12 mmu/-0.27 ppm), MH+: 884.55614 Da, RT: 26.9979 min,
Identified with: Sequest HT (v1.17); XCorr:3.25,
Fragment match tolerance used for search: 0.6 Da
Fragments used for search: -NH_3; y; b; y

b)

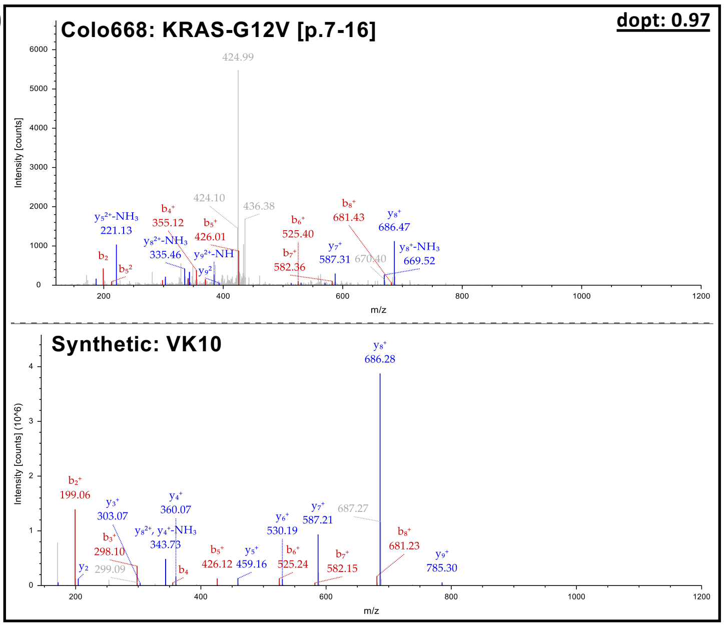

#1	b⁺	b²⁺	Seq.	y⁺	y²⁺	#2
1	100.07569	50.54148	V			10
2	199.14410	100.07569	V	785.48796	393.24762	9
3	298.21252	149.60990	V	686.41955	343.71341	8
4	355.23398	178.12063	G	587.35114	294.17921	7
5	426.27110	213.63919	A	530.32967	265.66847	6
6	525.33951	263.17339	V	459.29256	230.14992	5
7	582.36097	291.68412	G	360.22415	180.61571	4
8	681.42939	341.21833	V	303.20268	152.10498	3
9	738.45085	369.72906	G	204.13427	102.57077	2
10			K	147.11280	74.06004	1

Sequence: VVVGAVGVGK, Charge: +2, Monoisotopic m/z: 442.78180 Da (-0.03 mmu/-0.06 ppm), MH+: 884.55632 Da, RT: 26.7507 min,
Identified with: Sequest HT (v1.17); XCorr:1.82,
Fragment match tolerance used for search: 0.6 Da
Fragments used for search: -NH_3; y; b; y

#1	b⁺	b²⁺	Seq.	y⁺	y²⁺	#2
1	100.07569	50.54148	V			10
2	199.14410	100.07569	V	785.48796	393.24762	9
3	298.21252	149.60990	V	686.41955	343.71341	8
4	355.23398	178.12063	G	587.35114	294.17921	7
5	426.27110	213.63919	A	530.32967	265.66847	6
6	525.33951	263.17339	V	459.29256	230.14992	5
7	582.36097	291.68412	G	360.22415	180.61571	4
8	681.42939	341.21833	V	303.20268	152.10498	3
9	738.45085	369.72906	G	204.13427	102.57077	2
10			K	147.11280	74.06004	1

Sequence: VVVGAVGVGK, Charge: +2, Monoisotopic m/z: 442.78180 Da (-0.03 mmu/-0.06 ppm), MH+: 884.55632 Da, RT: 26.6971 min,
Identified with: Sequest HT (v1.17); XCorr:3.46,
Fragment match tolerance used for search: 0.6 Da
Fragments used for search: -NH_3; y; b; y

Supplementary Figure 12. Spectral validation for KRAS-G12V [p.7-16] in the Colo668 cell line by using a cognate synthetic peptide. a) To validate the reproducibility of KRAS-G12V [p.7-16] identification by global-immunopeptidomics, the Colo668 cell line, which has the same genetic background (KRAS-G12V somatic mutation with HLA-A*11:01) as ID172T, was analyzed by global-immunopeptidomics. MS2 spectra were obtained from an on-column volume of 6 μl (3.0e7 cells). **b)** Confirmation of KRAS-G12V [p.7-16] by targeted-immunopeptidomics. MS2 spectra obtained from an on-column volume of 1 μl (5e6 cells) are shown. To verify the MS2 spectra, we introduced the dotp score and confirmed the highly correlated dotp score between Colo668-derived KRAS-G12V [p. 7-16] (VVVGAVGVGK) and its cognate synthetic peptide, VK10. Gray peaks are the background noise, which is provably derived from irrelevant precursor ions.

Supplementary Figure 13

a) RCM1

Sample preparation from 1e8 RCM1 Cells
Sample dissolved in 20ul of Injection Buffer
MS on-column volume: 4ul (≒ 2.0e7 cells)/ CVSet)
Total: 6.0e7 cells
Analyses (3 CVSets) → Total: 9 FAIMS CVs

	Number
MS/MS	63867
PSMs	20819
Peptide Groups	7211
Protein Groups	3943

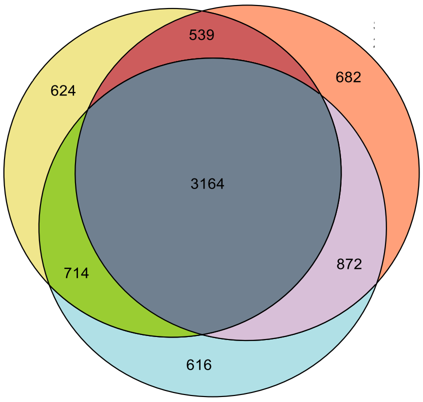

	Exclusive	Total	Label
A	624	5041	RCM1_4ul_CVSet1
B	682	5257	RCM1_4ul_CVSet2
C	616	5366	RCM1_4ul_CVSet3
A|B	539	3703	RCM1_4ul_CVSet1 | RCM1_4ul_CVSet2
B|C	872	4036	RCM1_4ul_CVSet2 | RCM1_4ul_CVSet3
A|C	714	3878	RCM1_4ul_CVSet1 | RCM1_4ul_CVSet3
A|B|C	3164	3164	RCM1_4ul_CVSet1 | RCM1_4ul_CVSet2 | RCM1_4ul_CVSet3
Sum	7211		

b)

A: CVSet1: by CV -40/-60/-80 V

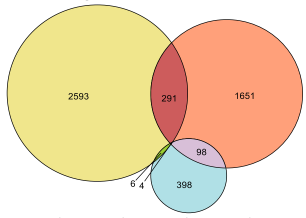

	Exclusive	Total	Label
A	2593	2894	Comp. Voltage [V]: -40
B	1651	2044	Comp. Voltage [V]: -60
C	398	506	Comp. Voltage [V]: -80
A|B	291	295	Comp. Voltage [V]: -40 | Comp. Voltage [V]: -60
B|C	98	102	Comp. Voltage [V]: -60 | Comp. Voltage [V]: -80
A|C	6	10	Comp. Voltage [V]: -40 | Comp. Voltage [V]: -80
A|B|C	4	4	Comp. Voltage [V]: -40 | Comp. Voltage [V]: -60 | Comp. Voltage [V]: -80
Sum	5041		

B: CVSet2: by CV -35/-50/-70 V

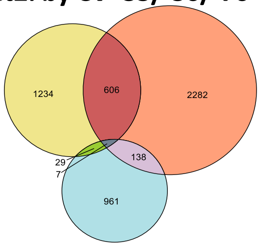

	Exclusive	Total	Label
A	1234	1876	Comp. Voltage [V]: -35
B	2282	3033	Comp. Voltage [V]: -50
C	961	1135	Comp. Voltage [V]: -70
A|B	606	613	Comp. Voltage [V]: -40 | Comp. Voltage [V]: -60
B|C	138	145	Comp. Voltage [V]: -60 | Comp. Voltage [V]: -80
A|C	29	36	Comp. Voltage [V]: -40 | Comp. Voltage [V]: -80
A|B|C	7	7	Comp. Voltage [V]: -40 | Comp. Voltage [V]: -60 | Comp. Voltage [V]: -80
Sum	5257		

C: CVSet3: by CV -45/-55/-65 V

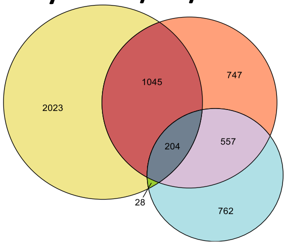

	Exclusive	Total	Label
A	2023	3300	Comp. Voltage [V]: -45
B	747	2553	Comp. Voltage [V]: -55
C	762	1551	Comp. Voltage [V]: -65
A|B	1045	1249	Comp. Voltage [V]: -40 | Comp. Voltage [V]: -60
B|C	557	751	Comp. Voltage [V]: -60 | Comp. Voltage [V]: -80
A|C	28	232	Comp. Voltage [V]: -40 | Comp. Voltage [V]: -80
A|B|C	204	204	Comp. Voltage [V]: -40 | Comp. Voltage [V]: -60 | Comp. Voltage [V]: -80
Sum	5366		

c)

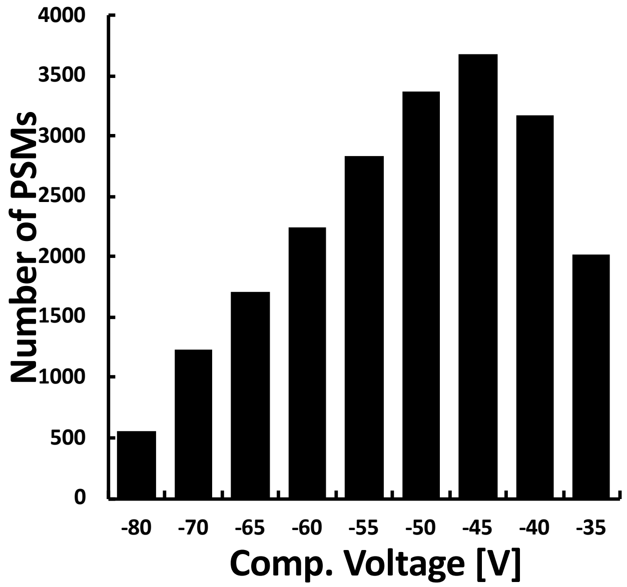

Supplementary Figure 13. Representative schematic diagram of global immunopeptidomics for RCM1. a) The number of cells used, FAIMS-assisted analytical conditions, counts of MS/MS, PSMs, peptide groups, protein groups and the overlap of identified peptide groups in each CVset with a Venn diagram are depicted. **b)** The three Venn diagrams show the breakdown of the number of peptide groups belonging to each CV. **c)** A bar graph depicts the PSM counts identified in each CV used in global-immunopeptidomics.

Supplementary Figure 14

a)

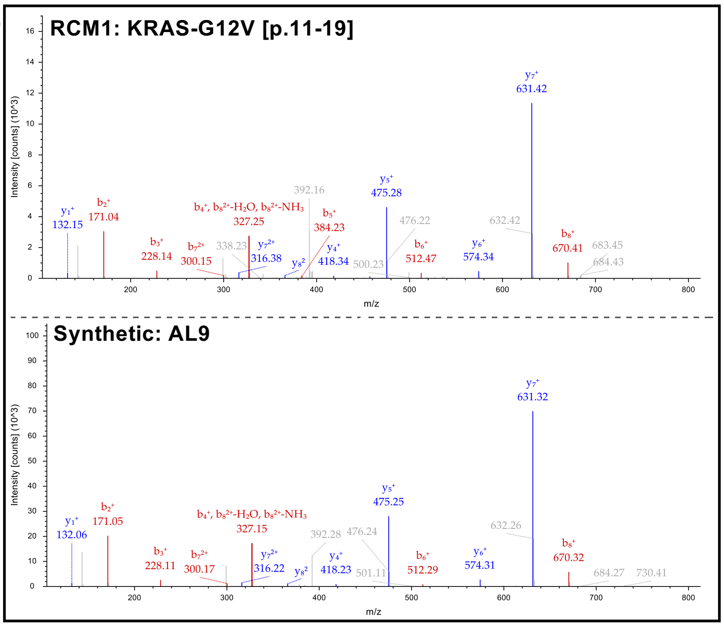

#1	b⁺	b²⁺	Seq.	y⁺	y²⁺	#2
1	72.04439	36.52583	A			9
2	171.11280	86.06004	V	730.44577	365.72652	8
3	228.13427	114.57077	G	631.37735	316.19231	7
4	327.20268	164.10498	V	574.35589	287.68158	6
5	384.22415	192.61571	G	475.28747	238.14738	5
6	512.31911	256.66319	K	418.26601	209.63664	4
7	599.35114	300.17921	S	290.17105	145.58916	3
8	670.38825	335.69776	A	203.13902	102.07315	2
9			L	132.10191	66.55459	1

Sequence: AVGVGKSAL, Charge: +2, Monoisotopic m/z: 401.24500 Da (-0.08 mmu/-0.21 ppm), MH+: 801.48271 Da, RT: 24.5948 min, Identified with: Sequest HT (v1.17); XCorr:2.12, Fragment match tolerance used for search: 0.6 Da
Fragments used for search: $-H_2O$; y; $-NH_3$; y; b; b; $-H_2O$; b; $-NH_3$; y

#1	b⁺	b²⁺	Seq.	y⁺	y²⁺	#2
1	72.04439	36.52583	A			9
2	171.11280	86.06004	V	730.44577	365.72652	8
3	228.13427	114.57077	G	631.37735	316.19231	7
4	327.20268	164.10498	V	574.35589	287.68158	6
5	384.22415	192.61571	G	475.28747	238.14738	5
6	512.31911	256.66319	K	418.26601	209.63664	4
7	599.35114	300.17921	S	290.17105	145.58916	3
8	670.38825	335.69776	A	203.13902	102.07315	2
9			L	132.10191	66.55459	1

Sequence: AVGVGKSAL, Charge: +2, Monoisotopic m/z: 401.24500 Da (-0.08 mmu/-0.21 ppm), MH+: 801.48271 Da, RT: 24.6681 min, Identified with: Sequest HT (v1.17); XCorr:2.51, Fragment match tolerance used for search: 0.6 Da
Fragments used for search: $-H_2O$; y; $-NH_3$; y; b; b; $-H_2O$; b; $-NH_3$; y

b)

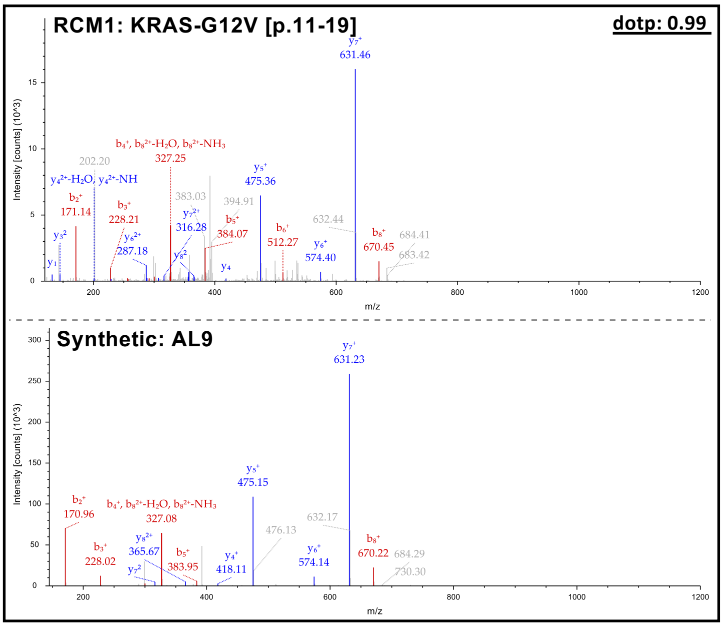

#1	b⁺	b²⁺	Seq.	y⁺	y²⁺	#2
1	72.04439	36.52583	A			9
2	171.11280	86.06004	V	730.44577	365.72652	8
3	228.13427	114.57077	G	631.37735	316.19231	7
4	327.20268	164.10498	V	574.35589	287.68158	6
5	384.22415	192.61571	G	475.28747	238.14738	5
6	512.31911	256.66319	K	418.26601	209.63664	4
7	599.35114	300.17921	S	290.17105	145.58916	3
8	670.38825	335.69776	A	203.13902	102.07315	2
9			L	132.10191	66.55459	1

Sequence: AVGVGKSAL, Charge: +2, Monoisotopic m/z: 401.24509 Da (+0.01 mmu/+0.02 ppm), MH+: 801.48290 Da, RT: 24.7408 min, Identified with: Sequest HT (v1.17); XCorr:1.91, Fragment match tolerance used for search: 0.6 Da
Fragments used for search: $-H_2O$; y; $-NH_3$; y; b; b; $-H_2O$; b; $-NH_3$; y

#1	b⁺	b²⁺	Seq.	y⁺	y²⁺	#2
1	72.04439	36.52583	A			9
2	171.11280	86.06004	V	730.44577	365.72652	8
3	228.13427	114.57077	G	631.37735	316.19231	7
4	327.20268	164.10498	V	574.35589	287.68158	6
5	384.22415	192.61571	G	475.28747	238.14738	5
6	512.31911	256.66319	K	418.26601	209.63664	4
7	599.35114	300.17921	S	290.17105	145.58916	3
8	670.38825	335.69776	A	203.13902	102.07315	2
9			L	132.10191	66.55459	1

Sequence: AVGVGKSAL, Charge: +2, Monoisotopic m/z: 401.24509 Da (+0.01 mmu/+0.02 ppm), MH+: 801.48290 Da, RT: 24.7448 min, Identified with: Sequest HT (v1.17); XCorr:1.97, Fragment match tolerance used for search: 0.6 Da
Fragments used for search: $-H_2O$; y; $-NH_3$; y; b; b; $-H_2O$; b; $-NH_3$; y

Supplementary Figure 14. Spectral validation for KRAS-G12V [p.11-19] in the RCM1 cell line by cognate synthetic peptide. a) Another KRAS-G12V-carrying immunopeptide (KRAS-G12V [p.11-19]) was identified by global-immunopeptidomics of RCM1. MS2 spectra obtained from an on-column volume of 4 μl (2.0e7 cells) are shown. **b)** Confirmation of KRAS-G12V [p.11-19] by targeted-immunopeptidomics. MS2 spectra obtained from an on-column volume of 4 μl (2.0e7 cells) are shown. To verify the MS2 spectra, we introduced the dotp score and confirmed the highly correlated dotp score between Colo668-derived KRAS-G12V [p.11-19] (AVGVGKSAL) and its cognate synthetic peptide AL9. Gray peaks are the background noise, which is provably derived from irrelevant precursor ions for identification.

Supplementary Figure 15

a)

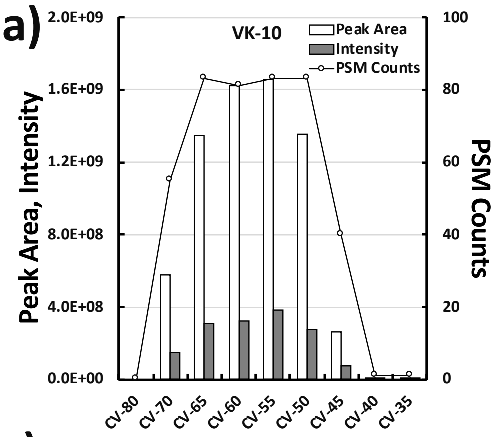

CV [V]	PSM Count
-80	0
-70	55
-65	83
-60	81
-55	83
-50	83
-45	40
-40	1
-35	1
**Sum**	**427**

b)

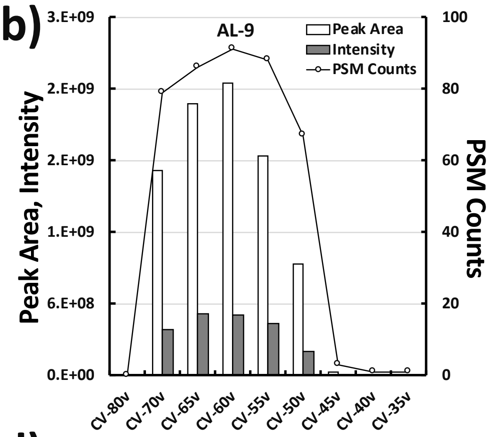

CV [V]	PSM Count
-80	0
-70	79
-65	86
-60	91
-55	88
-50	67
-45	3
-40	1
-35	1
**Sum**	**416**

c)

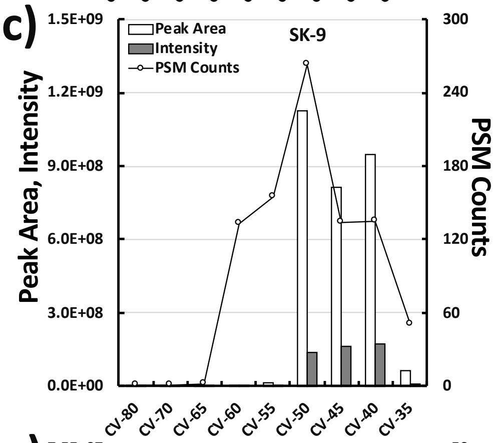

CV [V]	PSM Count
-80	1
-70	1
-65	2
-60	133
-55	155
-50	263
-45	134
-40	135
-35	51
**Sum**	**875**

d)

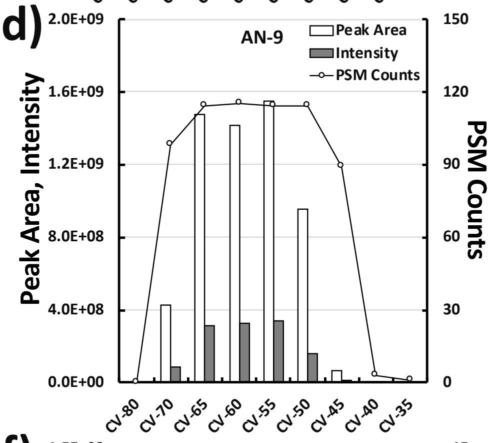

CV [V]	PSM Count
-80	0
-70	98
-65	114
-60	115
-55	114
-50	114
-45	89
-40	3
-35	1
**Sum**	**648**

e)

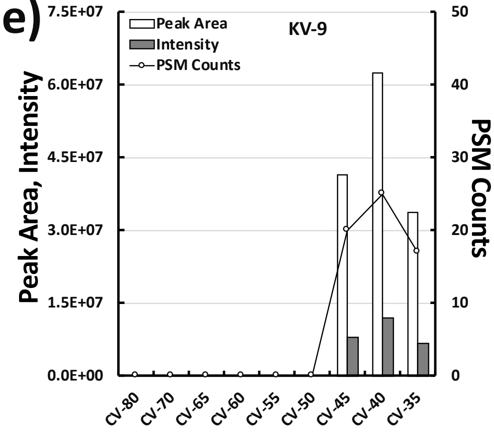

CV [V]	PSM Count
-80	0
-70	0
-65	0
-60	2
-55	5
-50	3
-45	5
-40	9
-35	0
**Sum**	**24**

f)

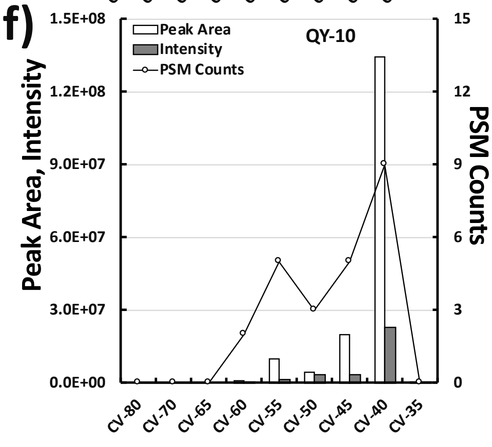

CV [V]	PSM Count
-80	0
-70	0
-65	0
-60	0
-55	0
-50	0
-45	20
-40	25
-35	17
**Sum**	**62**

g)

Sample Type	Sample	Origin of Neoantigen	Peptide Sequence	Name of Corresponding Synthetic Peptide	CVs ever confirmed in neoantigen identification
Tissue	ID172T	KRAS-G12V [p.7-16]	VVVGAVGVGK	VK10	-60v, -65v,
	ID261HT	CPPED1-R228Q	STQKKLADK	SK9	-45v
Cell Line	HCT116	GAPDH-I69T	AENGKLVTN	AN9	-50v, -55v, -60v, -65v, -70v
		NAPA-A181V	KVIDIYEQV	KV9	-35v, -40v, -45v
		CHMP7-A324T	QTDQMVFNTY	QY10	-40v, -45v, -50v, -55v
	Colo668	KRAS-G12V [p.7-16]	VVVGAVGVGK	VK10	-60v, -65v
	RCM1	KRAS-G12V [p.11-19]	AVGVGKSAL	AL9	-60v, -65v, -70v

Supplementary Figure 15. Distinct ion mobility of synthetic peptides assessed by FAIMS-assisted global-immunopeptidomics. 100 fmol of synthesized peptide, except for CHMP-A324T (approximately 72.5 fmol), were analyzed by global-immunopeptidomics. The intensity, peak area and corresponding PSM counts in each CV of **a**) KRAS-G12V [p.7-16]: VVVGAVGVGK, **b**) KRAS-G12V [p.11-19]: AVGVGKSAL, **c**) CPPED1-R228Q: STQKKLADK, **d**) GAPDH-I69T: AENGKLVTN, **e**) NAPA-A181V: KVIDIYEQV, and **f**) CHMP7-A324T: QTDQMVFNTY are depicted in a complex graph (left). The number of PSM counts is shown in the right table. The easily ionized peptides yielding hundreds of PSM counts were generally found in a broader range of CVs, while the ions with less ionization efficiency, yielding at most dozens of PSM counts, were found in a more restricted (narrow) range of CVs. **g)** A table shows the actual CVs used in neoantigen identification from related samples used in this study.

Supplementary Figure 16

a) **b)**

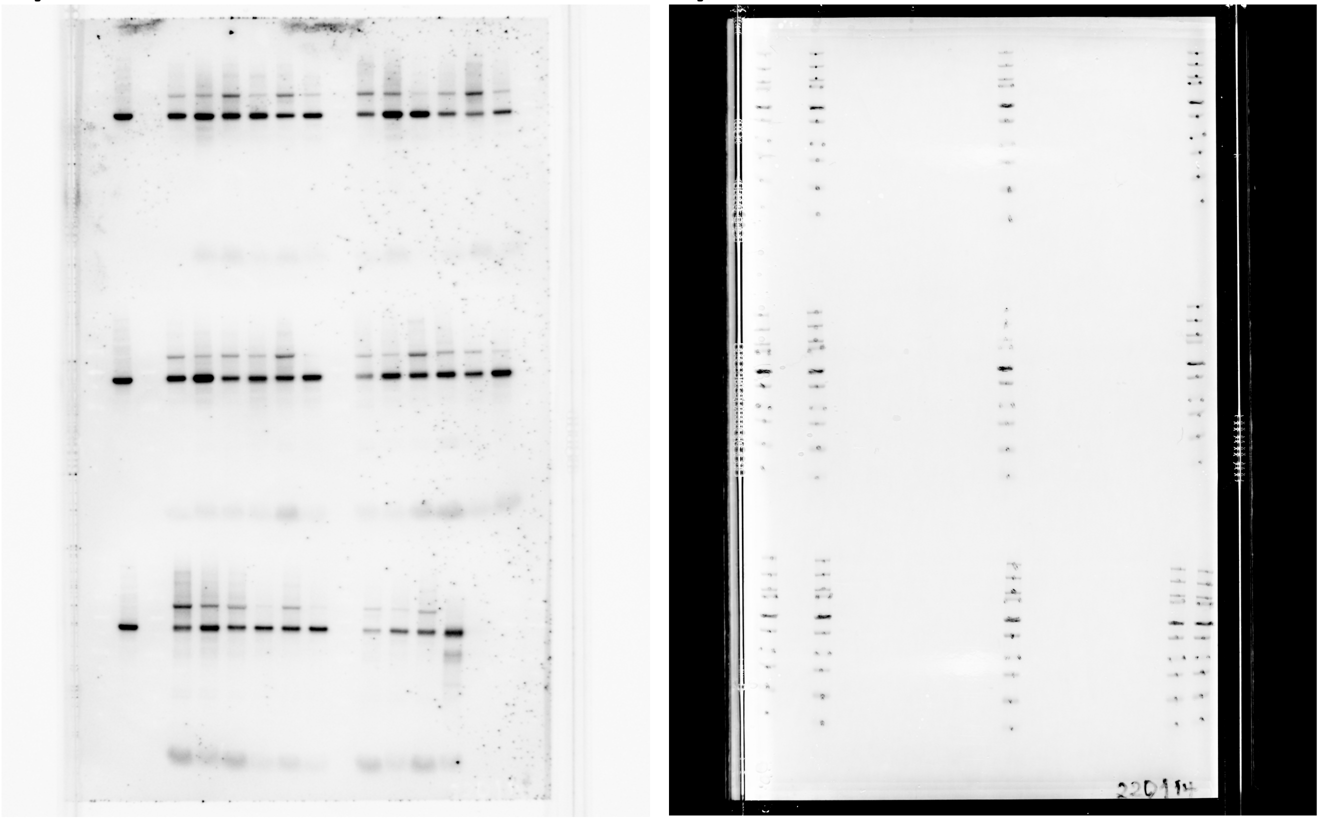

Supplementary Figure 16. The original images of Western blotting used in Supplementary Figure 7. a) The original image of the Western blot of HLA class I expression was assessed by anti-alpha-chain antibody (Clone: EMR8-5). b). The original image of Western blot for molecular marker detection of the identical membrane used in a).
