# Supplementary material for: Differential ion mobility mass spectrometry in immunopeptidomics identifies neoantigens carrying colorectal cancer driver mutations
Source: Commun Biol. 2022 Aug 18;5:831. doi: 10.1038/s42003-022-03807-w (PMC9388627; doi:10.1038/s42003-022-03807-w)
Supplement: Supplementary file 3 — Description of Additional Supplementary Files [file 42003_2022_3807_MOESM3_ESM.pdf]

Description of Additional Supplementary Files

File name: Supplementary Data 1

Description:

Supplementary Data 1a: Source data for Figure 1b, 1c and1d. Validation results of immunopeptidomics analyses without FAIMS by HCT116 cells.

Supplementary Data 1b: Source data for Figure 1b, 1c and 1d. Validation results of immunopeptidomics analyses with FAIMS by HCT116 cells.

Supplementary Data 1c: Source data for Figure 1e and 1f. Validation results of 3 independent immunopeptidomics analyses with FAIMS by 1e8 cells of HCT116 cells.

File name: Supplementary Data 2

Description:

Supplementary Data 2a: General information of clinical tissue samples used in this study.

Supplementary Data 2b: Mutation burden, genetic background of KRAS and the HLA allotype of CRC samples used in this study.

Supplementary Data 2c: Source data for Figure 2a to 2h. Numerical scores of CRC immunopeptidomics analyses.

Supplementary Data 2d: Source data for Figure 2i to 2l. Total number of unique immunopeptide and total number of source proteins in CRC samples.

Supplementary Data 2e: Source data for Supplementary figure 2b and 2c. Number of no-binders by NetMHCpan prediction for CRC immunopeptidome in this study

File name: Supplementary Data 3

Description:

Supplementary Data 3a: Source data for Figure 3a. Peptide counts for amino acid usage at pΩ.

Supplementary Data 3b: Source data for figure 3b, 3c and 3d. Differences of amino acid usage of cysteine at pΩ.

Supplementary Data 3c: Source data for figure 3e, 3f and 3g. Differences of amino acid usage of arginine at pΩ.

Supplementary Data 3d: Source data for figure 3h, 3i, 3j and 3k. Differences of amino acid usage of tryptophan at pΩ.
